# *Neisseria gonorrhoeae* Suppresses Dendritic Cell-Induced, Antigen-Dependent CD4 T Cell Proliferation

**DOI:** 10.1371/journal.pone.0041260

**Published:** 2012-07-23

**Authors:** Weiyan Zhu, Melissa S. Ventevogel, Kayla J. Knilans, James E. Anderson, Laurel M. Oldach, Karen P. McKinnon, Marcia M. Hobbs, Gregory D. Sempowski, Joseph A. Duncan

**Affiliations:** 1 Division of Infectious Diseases, Department of Medicine, University of North Carolina, Chapel Hill, North Carolina, United States of America; 2 Department of Medicine and Human Vaccine Institute, Duke University Medical Center, Durham, North Carolina, United States of America; 3 Department of Microbiology and Immunology, University of North Carolina, Chapel Hill, North Carolina, United States of America; 4 Department of Pharmacology, University of North Carolina, Chapel Hill, North Carolina, United States of America; 5 Lineberger Comprehensive Cancer Center, University of North Carolina at Chapel Hill, Chapel Hill, North Carolina, United States of America; Indian Institute of Science, India

## Abstract

*Neisseria gonorrhoeae* is the second most common sexually transmitted bacterial pathogen worldwide. Diseases associated with *N. gonorrhoeae* cause localized inflammation of the urethra and cervix. Despite this inflammatory response, infected individuals do not develop protective adaptive immune responses to *N. gonorrhoeae*. *N. gonorrhoeae* is a highly adapted pathogen that has acquired multiple mechanisms to evade its host's immune system, including the ability to manipulate multiple immune signaling pathways. *N. gonorrhoeae* has previously been shown to engage immunosuppressive signaling pathways in B and T lymphocytes. We have now found that *N. gonorrhoeae* also suppresses adaptive immune responses through effects on antigen presenting cells. Using primary, murine bone marrow-derived dendritic cells and lymphocytes, we show that *N. gonorrhoeae*-exposed dendritic cells fail to elicit antigen-induced CD4+ T lymphocyte proliferation. *N. gonorrhoeae* exposure leads to upregulation of a number of secreted and dendritic cell surface proteins with immunosuppressive properties, particularly Interleukin 10 (IL-10) and Programmed Death Ligand 1 (PD-L1). We also show that *N. gonorrhoeae* is able to inhibit dendritic cell- induced proliferation of human T-cells and that human dendritic cells upregulate similar immunosuppressive molecules. Our data suggest that, in addition to being able to directly influence host lymphocytes, *N. gonorrhoeae* also suppresses development of adaptive immune responses through interactions with host antigen presenting cells. These findings suggest that gonococcal factors involved in host immune suppression may be useful targets in developing vaccines that induce protective adaptive immune responses to this pathogen.

## Introduction

There are approximately 60 million cases of *N. gonorrhoeae* infection each year worldwide [1]. *N. gonorrhoeae* generally infects the female cervix or male urethra, where the local inflammatory response to mucosal invasion by the organism leads to symptoms of urethritis or cervicitis. Additionally, asymptomatic infection or colonization of mucosal surfaces with minimal inflammatory response occurs in approximately half of all infected individuals [2], [3]. *N. gonorrhoeae* infections significantly impact female reproductive health, as ascending infections of fallopian tubes are associated with infertility and perinatal infection can be transmitted to the neonate during birth. Furthermore, infection with *N. gonorrhoeae* is associated with increased risk of HIV transmission through effects on both HIV-infected and HIV-uninfected individuals. HIV-infected individuals with *N. gonorrhoeae* co-infection have increased levels of HIV virus in their blood, genital secretions, and semen [4], [5]. HIV-uninfected individuals with gonorrhea have increased numbers of inflammatory cells in their genital mucosa, some of which are susceptible to HIV infection, thereby increasing the risk that *N. gonorrhoeae*-infected individuals will acquire HIV from HIV-infected partners. Thus, prevention of *N. gonorrhoeae* infection is an important public health issue.

Despite experiencing localized inflammatory responses to *N. gonorrhoeae*, which can be very robust, most infected individuals do not develop protective adaptive immune responses to *N. gonorrhoeae*. This is clearly demonstrated by a high-frequency of recurrent infections caused by the same strain of *N. gonorrhoeae* in STD clinic patients [6]. Additionally, titers of anti-gonococcal antibodies are low and transient in patients with uncomplicated natural *N. gonorrhoeae* infection [7], [8] as well as experimentally-induced gonococcal urethritis [7], [8]. Mechanisms leading to this ineffective adaptive immune response are likely multifactorial including both antigenic variation of major surface molecules and active suppression of host immune signaling by this highly adapted human pathogen.

*Neisseria* species are known to induce inflammatory signaling in host cells through activation of innate pattern receptor molecules, including Toll-like receptors (TLR), TLR2 and TLR4, as well as C-lectin receptors, including dendritic-cell-specific ICAM-3 grabbing non-integrin [9], [10], [11]. *N. gonorrhoeae* also engages immunosuppressive signaling pathways in mammalian cells including B and T lymphocytes [12], [13]. A number of outer membrane adhesin proteins encoded by *opa* genes from *N. gonorrheoae* and *N. meningitidis* have been shown to engage host surface receptors known as carcinoembryonic antigen-related cellular adhesion molecules (CEACAMs) [14]. Ligation of CEACAM1 and CEACAM3 on human B and T cells by *N. gonorrhoeae* Opa proteins inhibits antibody production and cellular proliferation and can induce apoptosis [12], [15], [16], [17]. At high bacteria to T cell ratios, Opa-expressing *Neisseria* do not inhibit human T cell proliferation induced by mitogen exposure *in vitro* [18]. The gonococcal type IV pilus has also been implicated in T cell interaction. The gonococcal pilus has been shown to interact with CD46 and induce production of IL-10, an immunoregulatory cytokine. Piliated *N. gonorrhoeae* and isolated pilin also induce T cell proliferation [19]. The significance of direct effects of *N. gonorrhoeae* on T cells, through Opa-CEACAM and pilus-CD46 interactions, and the physiologic consequences of gonococcal engagement of this signaling system on infection, pathogenesis and immunity remain to be fully determined.

Recent studies have also demonstrated that immunologic response to *N. gonorrhoeae* is not only blunted but also skewed towards non-protective responses. In mice, infection with *N. gonorrheoae* induces differentiation of IL-17-producing CD4+ lymphocytes known as TH17 cells [20]. IL-17 levels are increased in both mice and humans infected with *N. gonorrheoae* [20], [21]. TH17 activation drives induction of localized inflammation including recruitment of host neutrophils, which is relatively ineffective in protecting against *N. gonorrhoeae* infection because the bacteria are relatively resistant to neutrophil mediated killing [22], [23]. Liu and Russell also demonstrated that blockade of host TGF-β during infection inhibits TH17 skewing and promotes the development of protective immune responses to *N gonorrhoeae*, suggesting that both inhibition and skewing of immunologic responses likely contribute to the host's inability to mount effective immunologic responses to this organism [24].

Prior to this report, the potential for *N. gonorrhoeae* to manipulate host immunologic response through antigen presenting cells, which act as the bridge between the innate and adaptive immune systems, was largely unexplored. We now show that *N. gonorrhoeae* potently inhibits the ability of antigen-primed dendritic cells to trigger T cell proliferation by inducing expression of both immunosuppressive cytokines and tolerance-inducing cell surface proteins.

## Results

### *N. gonorrhoeae* inhibits DC antigen-induced T cell proliferation

**Figure 1:**
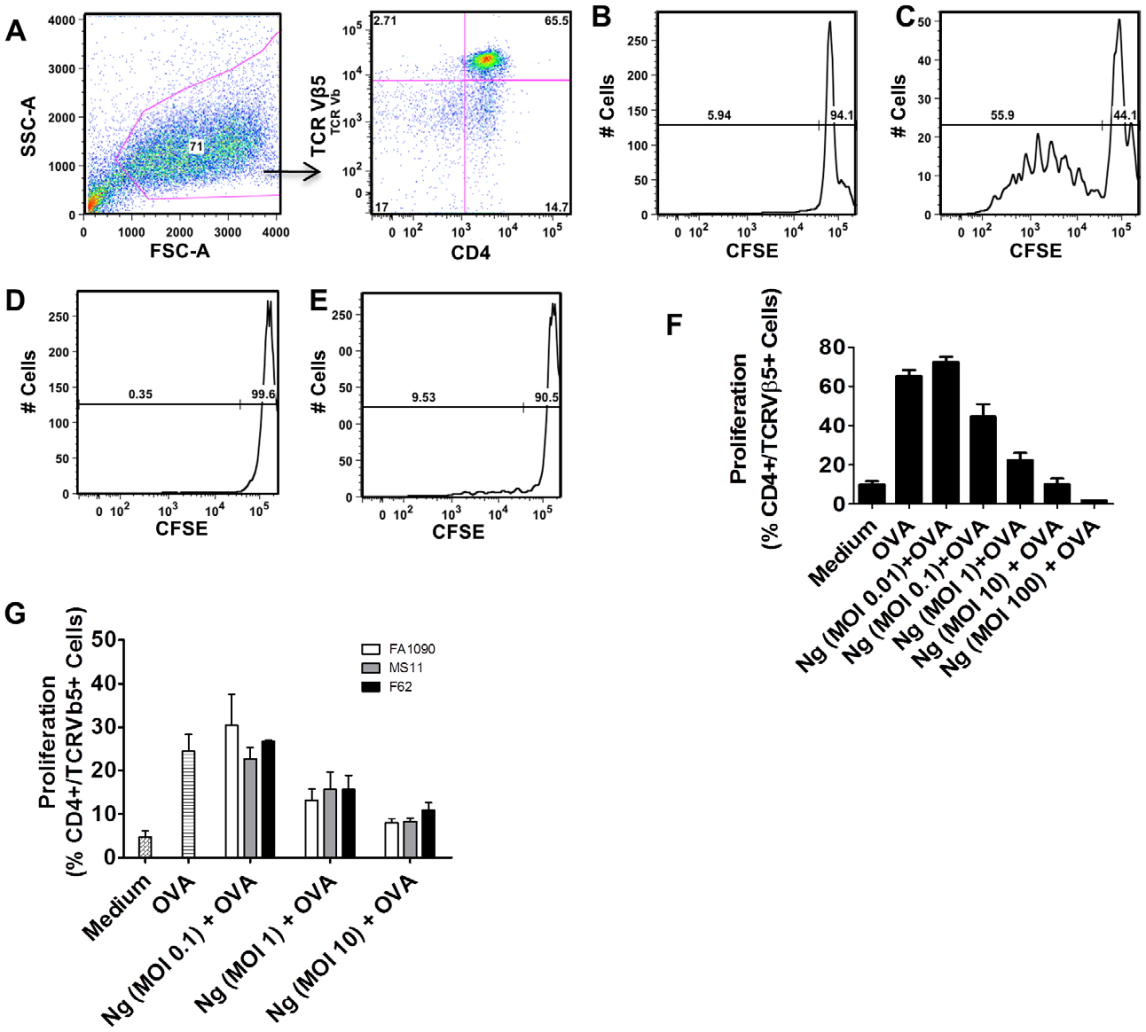
*N. gonorrhoeae* inhibits BMDC antigen-induced T cell proliferation. BMDCs were exposed to *N. gonorrhoeae* at different MOIs with or without OVA for 24 hours and then co-cultured with CFSE-loaded OT-II T cells for seven days. T cell proliferation to OVA was assessed by flow cytometric analysis. **A**) Representative gating strategy of CD4+ Vβ5+ OT-II T cells. **B**) Representative T cell proliferation following co-culture with medium only-treated BMDCs. **C**) Representative T cell proliferation following co-culture with OVA (100 µg/mL) pulsed BMDCs. **D**) Representative T cell proliferation profile following co-culture with *N. gonorrhoeae* (MOI = 1) exposed BMDCs. **E**) Representative T cell proliferation following co-culture with *N. gonorrhoeae* (MOI = 1) plus OVA (100 µg/mL) pulsed BMDCs. **F**) Percentage of OT-II T cell OVA-induced proliferation with a dose range of *N. gonorrhoeae* (0.01–10 MOI)-exposed BMDCs. Data are mean ± standard deviation (N = 8–32). G. OVA (100 µg/mL) pulsed BMDC were treated with different *N. gonorrhoeae* strains (White bars: FA1090; Gray bars: MS11; Black bars: F62) at the indicated doses (MOI 0.1–10). Antigen-induced T cell proliferation was assessed after co-culture of the *N. gonorrhoeae* and OVA treated BMDC with CFSE-loaded OT-II T cells for seven days as noted above. The percentages of proliferated T cells are plotted. Data are mean ± standard deviation (N = 3).

We sought to assess the effects of *N. gonorrhoeae* on antigen presenting cell-directed T cell proliferation. Because there is no known immunologic correlate of protection for gonorrhea, and the immunologic response to *N. gonorrhoeae* is weak in the human host, we sought to study the effect of gonococci on dendritic cell-T cell interactions in a system with a defined MHC class II-antigen-T-cell receptor combination. We co-cultured ovalbumin-treated murine C57BL6 bone marrow-derived dendritic cells (BMDC) with lymphocytes from OT-II mice. OT-II mice express a transgenic recombinant T cell receptor that recognizes amino acids 322 to 339 of the ovalbumin protein in the context of the MHC Class II I-A^2^ allele [25]. After seven days of differentiation, BMDCs were pulsed with ovalbumin, *N. gonorrhoeae* (strain FA1090), or the combination of both. To eliminate the possibility that *N. gonorrhoeae* might exert direct effects on the cultured T cells, extracellular *N. gonorrhoeae* in the culture medium were killed by addition of gentamicin after four hours, and gentamicin was left in the culture medium overnight. Quantitative culture of both medium and lysed dendritic cells confirmed that no viable *N. gonorrhoeae* were present 24 hours after antibiotic exposure (data not shown). Survival of dendritic cell-associated *N. gonorrhoeae* was measured after the initial four-hour incubation of *N. gonorrhoeae* and murine dendritic cells. At multiplicities of infection (MOI) of 1 or 10 cfu/dendritic cell, less than 1% of the initial *N. gonorrhoeae* inoculum was associated with dendritic cells (Figure S1). In dendritic cells exposed to *N. gonorrhoeae* at an MOI of ∼1, intracellular bacteria were not detectable even one hour after administration of extracellular gentamicin. In dendritic cells exposed to a higher inoculum, more than 99% of intracellular *N. gonorrhoeae* were eliminated by 1 hour, and no intracellular *N. gonorrhoeae* were detectable at 24 hours, even when extracellular gentamicin was removed from the culture (Figure S1). After 24 hours of exposure to ovalbumin or ovalbumin with *N. gonorrhoeae*, dendritic cells were washed to remove excess bacteria and bacterial products, and these cells were then co-cultured with carboxyfluorescein succinimidyl ester (CFSE)-labeled, enriched T lymphocytes from OT-II mice. Following seven days of co-culture with ovalbumin (OVA)-pulsed dendritic cells, OT-II transgenic T cells (CD4+,Vβ5+) showed significant proliferation, which was demonstrated by antigen-dependent dilution of CFSE-fluorescence in these cells (Figure 1 C) compared to cells exposed to dendritic cells treated with medium or *N. gonorrhoeae* in the absence of OVA (Figure 1 B and D). The T cell proliferative response to OVA-pulsed dendritic cells was essentially ablated by exposure of the DCs to *N. gonorrhoeae* (Figure 1 E). The inhibition of T cell proliferation was studied across a range of bacterial concentrations (MOI from 0.1 to 100 cfu/dendritic cell) and was found to be quite potent, with a noticeable effect often seen with a ratio of bacteria to dendritic cells as low as 1 to 10 (MOI 0.1). Nearly complete inhibition was usually noted at MOI of 1.0 (Figure 1 E). Similar inhibitory effects were noted when dendritic cells were treated with *N. gonorrhoeae* strain MS11 or F62 (Figure 1G). A similar lack of proliferative response to antigen was noted when an ovalbumin peptide was expressed in a surface–exposed loop of the *N. gonorrhoeae* outer-membrane protein OpaB, demonstrating that inhibition was not an artifact of co-administration of exogenous antigen and bacteria (Figure S2) [26]. These results suggest that *N. gonorrhoeae* exerts an immunosuppressive effect on CD4 T cells through antigen presenting cells that are exposed to the bacteria.

**Figure 2:**
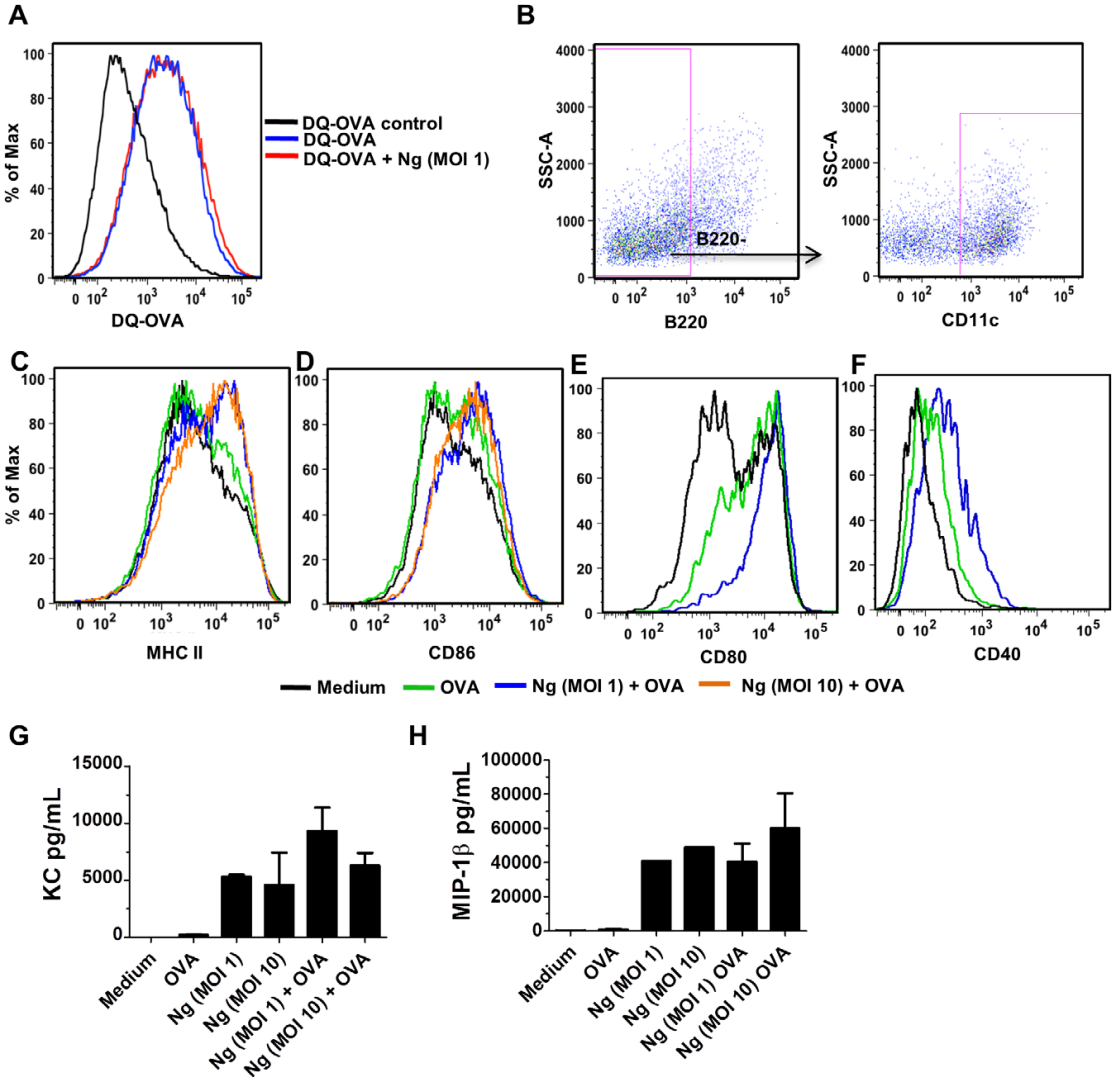
*N. gonorrhoeae* does not impact OVA uptake and processing by BMDCs, but does induce maturation and co-stimulatory molecule expression and inflammatory cytokine/chemokine production on BMDCs. **A**) Flow cytometric analysis of DQ-OVA endocytosis by BMDCs exposed to *N. gonorrhoeae* versus control (MOI = 1). **B**) Gating strategy of BMDCs used for surface marker expression analysis (B220− CD11c+). **C–F**) Representative histograms from 3–4 independent experiments showing BMDC expression of CD80, CD86, CD40 and MHC class II 24 hours post stimulation with medium only, OVA (100 µg/mL), or *N. gonorrhoeae* (MOI = 1, 10) with OVA (100 µg/mL). BMDCs were exposed to *N. gonorrhoeae* at MOI of 1 or 10, alone or with OVA and cytokine and chemokine secretion was determined using multiplex bead-based assay analysis of the culture supernatants as described in the experimental procedures. **G**) KC, **H**) MIP-1β Data shown are mean ± standard deviation (N = 4).

Several lines of investigation were pursued to better define the mechanism by which *N. gonorrhoeae* exerted this anti-proliferative effect on T cells cultured with antigen-pulsed DCs. To determine whether *N. gonorrhoeae* was simply blocking the uptake or processing of ovalbumin antigen in the murine DCs, DCs were incubated with ovalbumin covalently linked to both a fluorescent reporter and a fluorescent quenching molecule (DQ-OVA). When DCs take up and proteolytically process DQ-OVA, they exhibit a characteristic shift in fluorescence intensity (Figure 2 A). When *N. gonorrhoeae* was added to the culture medium with DQ-OVA, uptake and processing of DC-OVA by the dendritic cells, measured by flow cytometry, was not altered (Figure 2 A). Dendritic cells were clearly recognizing *N. gonorrhoeae*, as they robustly secreted proinflammatory cytokines and chemokines including KC (the murine equivalent of IL-8) and MIP1β after treatment with *N. gonorrhoeae* for 24 hours at MOI of 1 or 10. Antigen presentation and induction of T cell proliferation by DCs requires expression of both MHC class II and co-stimulatory surface molecules that interact with receptors on T cells, including CD80, CD86, and CD40 [27]. DCs that were pulsed with ovalbumin and *N. gonorrhoeae* upregulated expression of each of these co-stimulatory molecules equal to or greater than upregulation seen after exposure to ovalbumin alone. Additionally, expression of both MHC class II and CD86 increased with increasing doses of *N. gonorrhoeae* while the ability of the dendritic cells to stimulate T-cell proliferation was decreased (Figure 2 C–F).

Overall, these data indicate that *N. gonorrhoeae* does not prevent antigen-pulsed DCs-induced T cell proliferation by blockade of DC maturation, expression of MHC class II or co-stimulatory molecules. IL-2 is an important cytokine in T cell proliferation that is initially expressed by DCs and later by stimulated T cells [27]. Despite dramatic reductions in proliferating T cells, the levels of IL-2 in culture supernatants from DC-T cell co-cultures were equivalent to levels from co-cultures with DCs pulsed with ovalbumin or ovalbumin with *N. gonorrhoeae* (Figure S3).

### *N. gonorrhoeae* inhibits DC-mediated, antigen-induced T cell proliferation through multiple mechanisms

**Figure 3:**
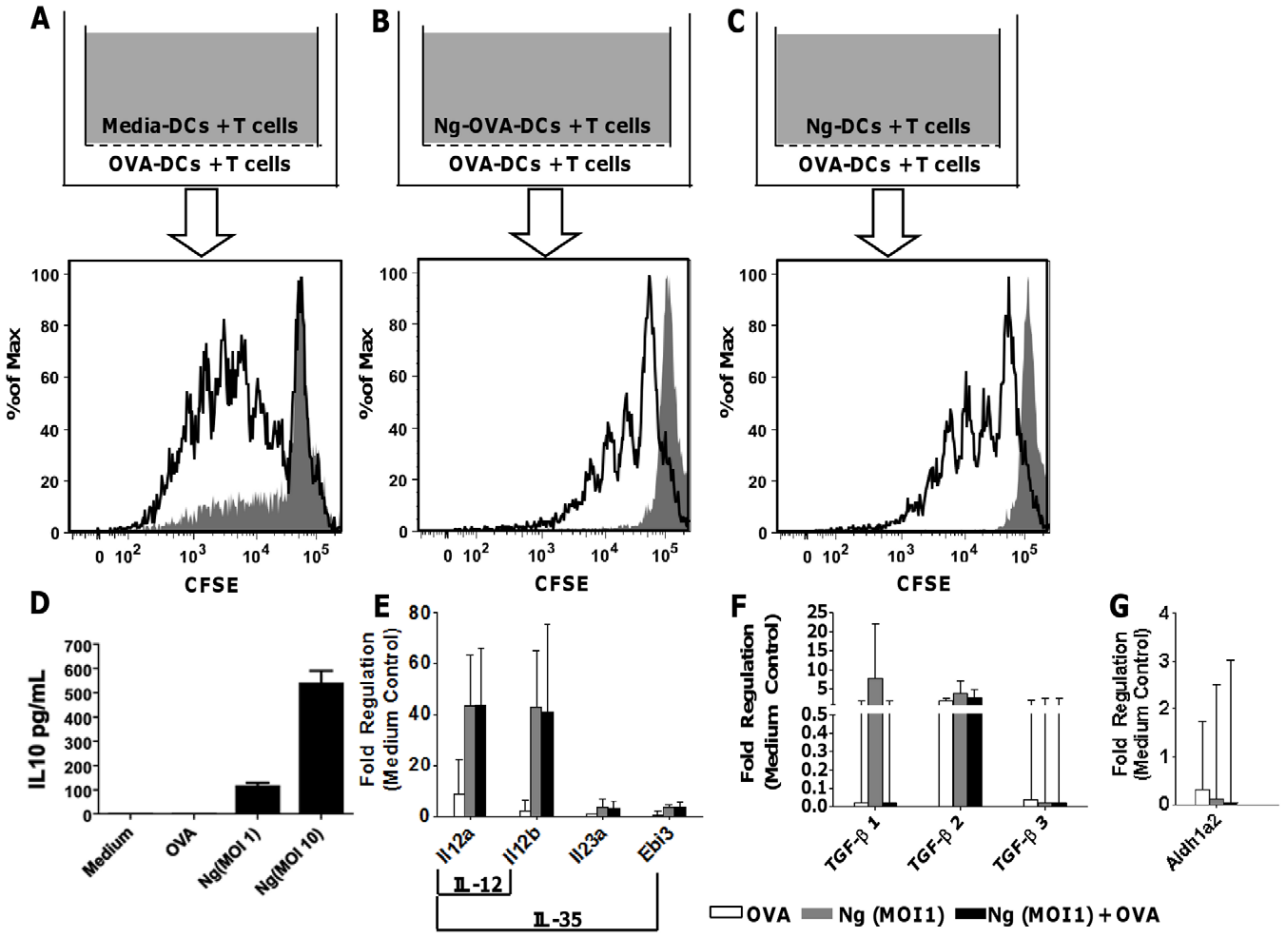
Soluble factors in BMDC/T cell co-culture partially inhibit OVA-induced T cell proliferation. **A–C**) CFSE proliferation profiles for OT-II T cells co-cultured with BMDCs under indicated conditions. Representative CFSE profiles for T cells from transwell insert (gray) and transwell itself (open) are shown (from three independent experiments). **D**) IL-10 protein production by BMDCs cultured with Medium, OVA, *N. gonorrhoeae* (MOI = 1, 1). Mean pg/mL ± SD, N = 3. **E**) *Il12a*, *Il12b*, *Il23a* and *Ebi3* mRNA steady-state expression in BMDCs cultured with OVA, *N. gonorrhoeae* (MOI = 1), or *N. gonorrhoeae* (MOI = 1) with OVA. Mean fold regulation ± SD, N = 3. **F**) Steady-state expression of mRNA encoding TGF-β 1, 2 and 3 in BMDCs cultured with OVA, *N. gonorrhoeae* (MOI = 1), or *N. gonorrhoeae* (MOI = 1) plus OVA. Mean fold regulation ± SD, N = 3. **G**) *Aldh1a2* mRNA steady-state expression in BMDCs cultured with OVA, *N. gonorrhoeae* (MOI = 1), or *N. gonorrhoeae* (MOI = 1) with OVA. Mean fold regulation (decrease) ± SD, N = 3.

We next determined whether *N. gonorrhoeae*-treated DCs inhibited T cell proliferation through secretion of soluble factors. DCs pulsed with ovalbumin and/or *N. gonorrhoeae* were co-cultured with OT-II lymphocytes in transwells, with a pore size of 0.4 µm, to allow diffusion of soluble factors while preventing translocation of dendritic cells between cultures (Figure 3 A–C). Whereas co-incubation with *N. gonorrhoeae* completely abrogated T cell proliferation in wells occupied by *N. gonorrhoeae*-treated DCs, there was only partial inhibition of ovalbumin-pulsed DC induced-T cell proliferation in the inserted transwell. These data suggest that soluble factors produced by *N. gonorrhoeae*-treated dendritic cell-T cell co-culture contributed to inhibition of T cell proliferation, but these soluble factors were insufficient to recapitulate the entire inhibitory effect.

We examined DC production of known immunomodulatory cytokines IL-10, IL-35, and TGF-β after treatment with *N. gonorrhoeae*. Secreted IL-10 protein was detected in supernatants of dendritic cell cultures treated with *N. gonorrhoeae* or *N. gonorrhoeae* with ovalbumin (Figure 3 D). IL-35 protein was undetectable by ELISA in dendritic cell culture supernatants (data not shown). IL-35 is comprised of two subunits, the α chain of IL-12 (encoded by *Il12a*) and the Epstein Barr virus induced gene-3 (*Ebi3*). While mRNA for *Il12a* was greatly upregulated by *N. gonorrhoeae* treatment, *Ebi3* upregulation was modest at the steady-state mRNA level (Figure 3 E). mRNA encoding TGF-β1, TGF-β2, and TGF-β3 were not induced in *N. gonorrhoeae*-treated dendritic cells relative to ovalbumin treated dendritic cells (Figure 3 F). Because TGF β exerts immunomodulatory effects in the murine gonococcal infection model [24], these results suggest that TGF-β is either being produced in response to *N. gonorrhoeae* by other cell types in the genital tract or that it is produced constitutively in the antigen presenting cells, and other factors induced by *N. gonorrhoeae* exposure work in conjunction with TGF-β to suppress immune responses to this pathogen.

In addition to secreted cytokines, dendritic cells are also known to produce small molecules (e.g. retinoic acid) that promote immunosuppressive T regulatory cell production. The synthesis of retinoic acid is regulated by expression of the retinaldehyde dehydrogenase enzyme (RALHD2, encoded by the *Aldh1a2* gene) [28]. *N. gonorrhoeae*-treated dendritic cells did not show any significant change in *Aldh1a2* mRNA (Figure 3 G).

**Figure 4:**
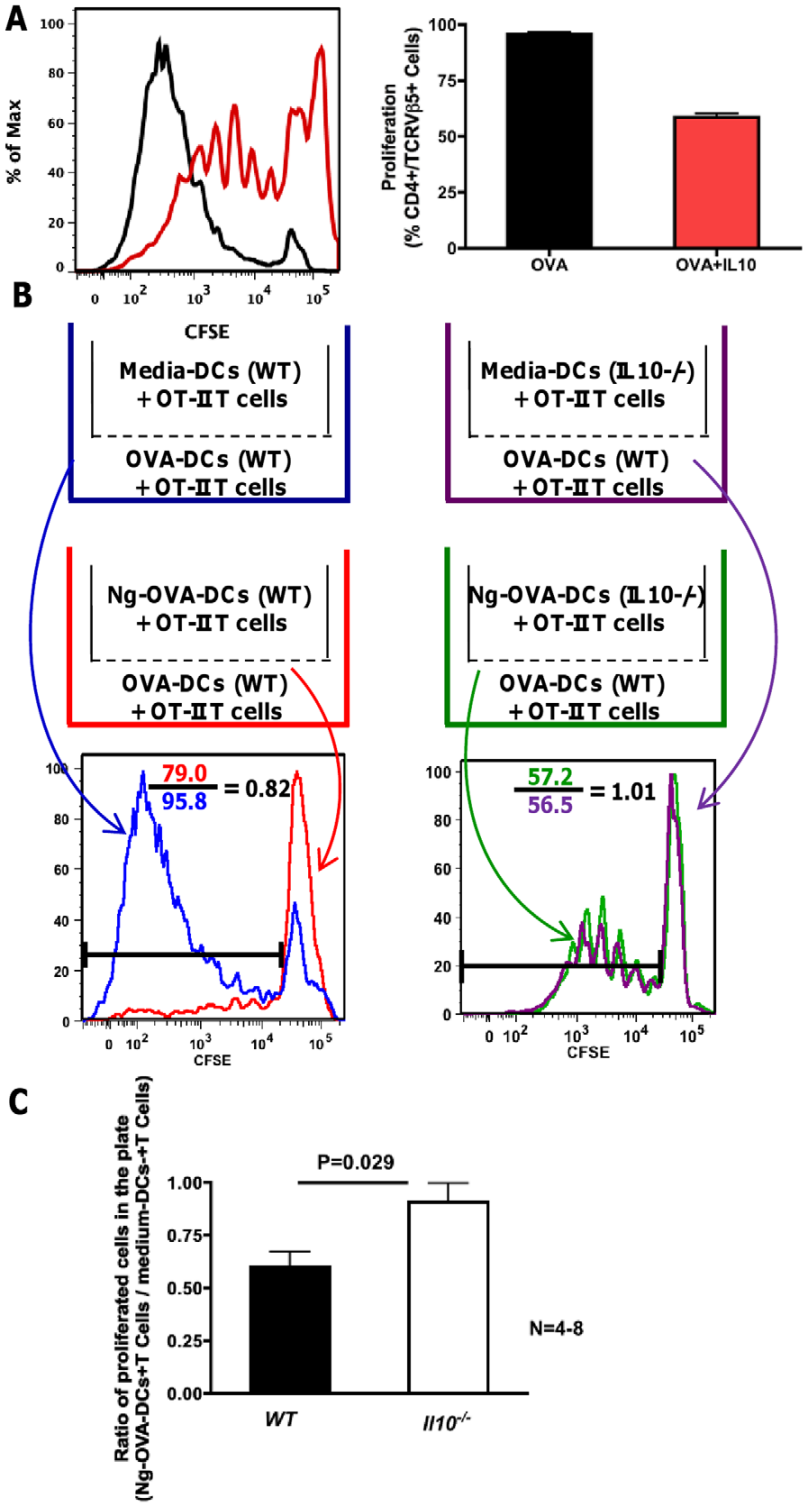
IL-10 inhibits OVA-DC-induced T cell proliferation. OVA-pulsed dendritic cells were co-cultured with CFSE-loaded OT-II T cells with or without IL-10 for seven days. **A**) Representative histogram overlay and bar graph show T cell proliferation profiles following culture with OVA-pulsed DCs (black) or OVA-pulsed DCs+IL-10 (red). The bar graph shows the proliferation of OT-II T cell in the presence of OVA-pulsed DCs with and without exogenous IL-10 from three independent experiments. Data are mean ± standard deviation (N = 3). **B**) Transwell experiment scheme. WT OVA-DC with OT-II T cell co-culture was placed in all transwell plates. In the insert medium treated-DCs or *N. gonorrhoeae*-treated DCs from wild type or *Il10^−/−^* were co-cultured with OT-II T cells as indicated. T cell proliferation from the transwell plate is shown in the histogram overlays. OVA-induced T cell proliferation in the plate was inhibited by *N. gonorrhoeae*-treated wild type DCs in the insert (red) but not by wild type medium treated-DCs in the insert (blue). OVA-induced T cell proliferation in the plate was the same for *N. gonorrhoeae*-treated *Il10^−/−^* DCs in the insert (green) and medium treated *Il10^−/−^* DCs in the insert (purple). **C**) Ratio of proliferated T cells from transwell plates with inserts supplying *N. gonorrhoeae*-OVA-DCs or medium-DCs. Ratio of T cell proliferation in the plate was obtained by dividing the *N. gonorrhoeae*-OVA-DCs insert by medium-DCs insert. The black bars represent proliferation ratio from transwell plate supplied with wild type BMDCs in insert (N = 8), the open bars represent proliferation ratio from transwell plate supplied with *Il10^−/−^* BMDCs in insert (N = 4).

We sought to further characterize the role of IL-10 secretion in the *N. gonorrhoeae*-induced suppression of dendritic cell-mediated T cell proliferation. First, purified, recombinant IL-10 was added to OVA-pulsed dendritic cells during antigen exposure as well as co-culture with OT-II T cells. This treatment did reduce T cell proliferation, indicating that IL-10 was capable of the suppressive effect observed in these cultures (Figure 4A). Second, dendritic cells derived from mice with a genetically disrupted *Il10* gene (*Il10^−/^* ^−^) were treated with *N. gonorrhoeae* and cultured in transwells adjacent to OVA-pulsed dendritic cell/OT-II T cell co-cultures, as described in figure 4B. *N. gonorrhoeae*-treated dendritic cells from *Il10^−/−^* mice did not inhibit T cell proliferation in adjacent transwells as well as wild type control dendritic cells treated with *N. gonorrhoeae* (Figure 4 B and C). Overall, these results indicate that IL-10 is upregulated in dendritic cells after *N. gonorrhoeae* exposure, and that IL-10 released by *N. gonorrhoeae*-treated dendritic cells is one of the soluble factors that inhibits T cell proliferation. Interestingly, OVA-induced proliferation was equally inhibited by *N. gonorrhoeae* treatment of wild-type C57/BL6 and *Il10^−/−^* BMDCs (Figure S4). These data, combined with the fact that transwell experiments demonstrate that dendritic cell-derived soluble factors alone are insufficient to fully mediate *N. gonorrhoeae*-induced suppression of T cell proliferation in this co-culture system, indicate that surface inhibitory factors, and possibly other soluble inhibitory factors, can compensate for the loss of BMDC IL-10. Therefore, we sought to determine whether *N. gonorrhoeae* induced expression of known inhibitory cell surface molecules on dendritic cells.

**Figure 5:**
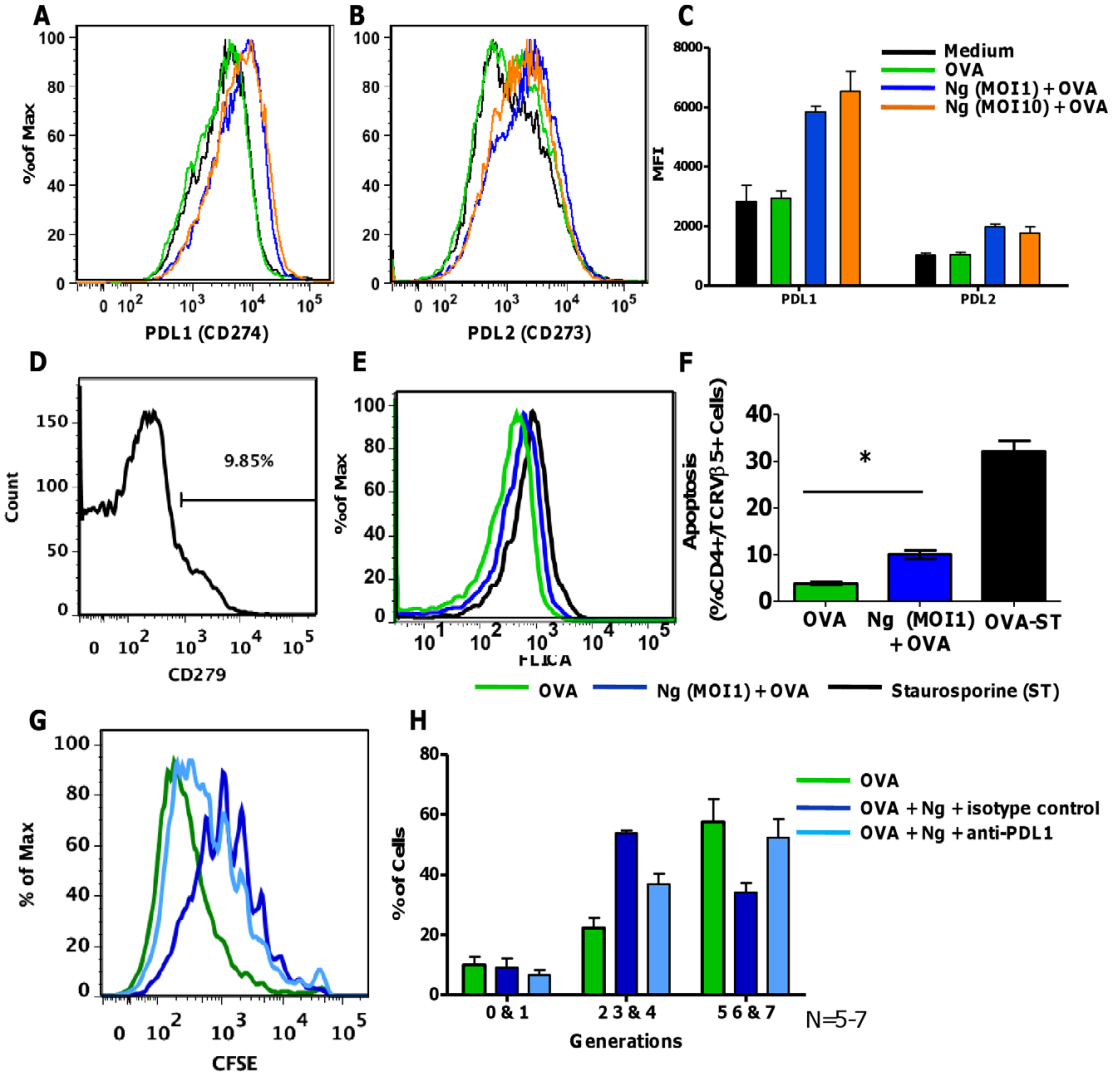
PD-L1 and PD-L2 are induced on *N. gonorrhoeae* exposed BMDCs. BMDCs treated for 24 hours with medium only, OVA, *N. gonorrhoeae* (MOI = 1,10) with OVA were immunostained for flow cytometric analysis of CD273 and CD274 on DCs (B220−, CD11c+). Representative overlay histograms of: **A**) CD274 (PD-L1) and **B**) CD273 (PD-L2). **C**) Median fluorescence intensity (MFI) of PD-L1 and PD-L2 expression on BMDCs treated as indicated. **D**) Histogram of PD1 (CD279) expression on CD4+ Vβ5+ OT-II T cells prior to co-culture with BMDCs. **E–F**) Caspase 3&7 activity (FLICA) form CD4+ Vβ5+ OT-II T cells following co-culture with OVA or *N. gonorrhoeae* (MOI = 1) plus OVA (100 µg/mL) pulsed BMDCs. **E**) Representative overlay histograms of Caspase 3&7 activity (FLICA) from CD4+ Vβ5+ OT-II T cells following co-cultured with BMDCs for 24 hours. **F**) Percentage of apoptotic CD4+ Vβ5+ OT-II T cells following co-cultured with BMDCs for 24 hours. Data are mean ± standard deviation (N = 4 replicates). T cells treated with 1 µM staurosporine (ST) for 3 hours was used as positive control. **G**) Representative overlay histograms of OT-II T cell proliferation induced by BMDCs treated with OVA (green) versus *N. gonorrhoeae* (MOI = 0.1) with OVA plus anti-PD-L1 (1∶10 dilute, light blue), *N. gonorrhoeae* (MOI = 0.1) with OVA plus isotype control (1∶10 dilute, dark blue). **H**) Mean % ± SD of OT-II T cells proliferated through generations 0–1, 2–4, 5–7 following indicated culture conditions, N = 5–7.

Expression of several DC surface proteins known to induce T cell anergy or tolerance was determined after treatment with *N. gonorrhoeae*. Some tolerogenic DCs express Programmed Death Ligand 1 and 2 (PD-L1 and PD-L2), which can activate the receptor, Programmed Death-1 (PD1), found on activated T cells to promote differentiation of immunosuppressive T regulatory cells [29]. DCs treated with *N. gonorrhoeae* with or without ovalbumin upregulated surface expression of both PD-L1 and PD-L2 (Figure 5 A–C). Using flow cytometry, PD1 was detected on the surface of about 10% (10.11+/−2.4%, N = 3) of CD4+ T cells used in the co-culture system (Figure 5 D). Activation of PD1 by PD-L1 and PD-L2 can induce apoptosis of activated T cells as well as promote anergy [30]. Consistent with this mechanism of action, increased activation of the apoptotic proteinase Caspase-3 was observed in T cells incubated with *N. gonorrhoeae*-treated DCs (Figure 5 E and Figure S5). Inhibition of T cell proliferation by *N. gonorrhoeae* was tested in the presence of anti-PD-L1 neutralizing antibodies or an isotype antibody control. Addition of PD-L1 neutralizing antibody to *N. gonorrhoeae*-treated, OVA-pulsed dendritic cells partially restored their ability to stimulate T cell proliferation (Figure 5 G and H). Other surface molecules that have been implicated in tolerogenic responses include ILT3/4 and ICOSL. Steady-state levels of mRNA encoding ILT3 and ICOSL were not modulated by exposure to *N. gonorrhoeae* (data not shown) [31], [32]. These data indicate that *N. gonorrhoeae* promoted up-regulation of at least two immunomodulatory surface proteins (PD-L1 and PD-L2). Further, *N. gonorrhoeae*-induced expression of PD-L1 is capable of attenuating antigen-specific CD4 T cell responses. In total, it appears that *N. gonorrhoeae* blocks DC induction of T cell proliferation through multiple mechanisms. This may represent functional redundancy or may indicate that significant suppression of the immune system requires cumulative effects on several regulatory pathways.

### *N. gonorrhoeae* induces tolerogenic responses in human DC

**Figure 6:**
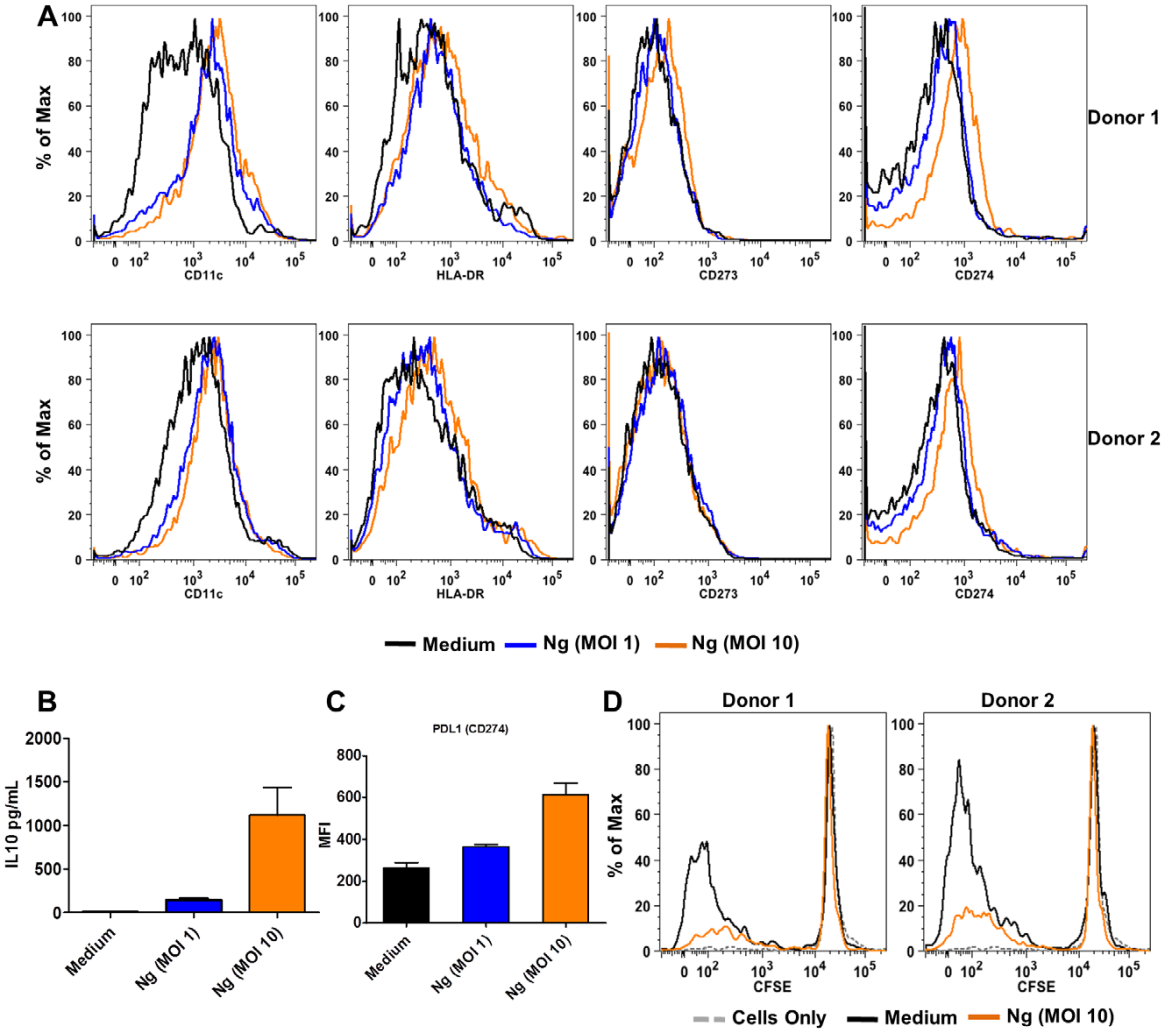
*N. gonorrhoeae* inhibits dendritic cell-induced T cell proliferation in human primary immune cells. **A**) Representative histograms from 2 donors showing unregulated expression of CD11c, HLA-DR, CD274 and CD273 at 24 hours post stimulation with *N. gonorrhoeae* (MOI = 1, 10). **B**) IL-10 protein production by human DCs treated with *N. gonorrhoeae* (MOI = 1,10). **C**) MFI of PD-L1 expression on human DCs treated with *N. gonorrhoeae* (MOI = 1,10). **D**) *N. gonorrhoeae* inhibits human DCs induced allogeneic T cell proliferation in the Mixed Lymphocyte Reaction (MLR). CFSE proliferation profiles of CD4+ cells after non-adherent cells (NAD) co-cultured with human DCs treated with medium or *N. gonorrhoeae* (MOI = 10) for 7 days at the ratio of 10∶1.

Because mice are not natural hosts of *N. gonorrhoeae*, we sought to determine whether *N. gonorrhoeae* could also induce PD-L1 expression and IL-10 secretion in human dendritic cells. Primary human dendritic cells were generated by *in vitro* differentiation of CD34+ cells from peripheral blood for 14 days. These cultured cells upregulated HLA-DR, CD11c in response to *N. gonorrhoeae* treatment (Figure 6 A), just as we observed in murine BMDC. Under these conditions, primary human dendritic cells also secreted IL-10 and upregulated surface PD-L1 expression (Figure 6 B and C). *N. gonorrhoeae*-treated human DC were co-cultured with heterologous CFSE labeled lymphocytes and other non-adherent cells. As observed in the murine co-culture system, *N. gonorrhoeae*-treatment inhibited the ability of dendritic cells to stimulate CD4+ T cell proliferation (Figure 6 D). Thus, *N. gonorrhoeae* inhibits murine and human dendritic cell-induced T cell proliferation.

## Discussion

It has long been recognized that *N. gonorrhoeae* has the capacity to modulate host immunologic responses to prevent protective adaptive immunity. Though T and B lymphocyte populations are generally responsible for mediating adaptive immunity, dendritic cells serve as the primary host cell involved in presenting pathogen-derived molecules to lymphocytes in order to generate adaptive immune responses. Both stimulatory and inhibitory effects have been observed in T and B lymphocyte function following exposure to *N. gonorrhoeae*. However, the effects of *N. gonorrhoeae* on lymphocyte function through antigen presenting cells, dendritic cells in particular, has not previously been reported. Recent studies by Russell and colleagues demonstrate that *N. gonorrhoeae* drives CD4+ T cell differentiation *in vivo* and *in vitro* towards the TH17 lineage [20], [24]. This process is dependent on host TGF-β, and inhibition of this cytokine permits the *in vivo* development of protective TH1 and TH2 immunologic responses in a murine model of *N. gonorrhoeae* [24]. Here we showed that, in addition to skewing the CD4+ T helper cell phenotype in a TGF-β dependent fashion, *N. gonorrhoeae* can also inhibit antigen specific CD4+ T cell proliferation through effects on host dendritic cells. The majority of experimental evidence is from murine immune cells because of the immunologic tools available for studying antigen specific stimulation and homogeneity of the host cells from inbred mouse strains. In addition, we showed that *N. gonorrhoeae* also mediates similar effects in human cells, highlighting the likely clinical relevance of these findings. The effects of *N. gonorrhoeae* on antigen-dependent T cell proliferation are not unlike those reported for *Lactobacillus*, a commensal organism of the lower female genital tract [33]. *N. gonorrhoeae* closely resembles other commensal *Neisseria* species in morphology and genetic make-up [34]. Interestingly, the gonococcus actually often appears to behave as a commensal in the setting of asymptomatic colonization of the vaginal mucosa, which occurs in over half of female patients with gonorrhea [35]. It is therefore not surprising that *N. gonorrhoeae* suppresses host adaptive immune responses that might aid the host in clearing bacteria through interactions with antigen presenting cells.

We found that *N. gonorrhoeae* inhibition of antigen-induced proliferations appears to result from modulation of multiple host factors. *N. gonorrhoeae* induced production of cell surface molecules on DCs that block T cell proliferation including PD-L1 and PD-L2. PD-L1 is known to play a role in reproductive tract immune tolerance, where its expression is critical to maternal fetal tolerance [36]. Further, PD-L1-mediated immunosuppression plays a role in response to other bacteria in the genital tract. *Lactococcus lactis*, a vaginal commensal bacterium, also activates tolerogenic, PD-L1-expressing dendritic cells [37]. Similarly, PD-L1 expression actually protects the upper genital tract from inflammatory damage in murine models of chlamydial infection [38]. We have now found that blockade of PD-L1 partially reverses gonococcal inhibition of T cell proliferation, confirming the notion that PD-L1 is involved in *N. gonorrhoeae*-induced immunomodulation. However, the partial reversal suggests contributions by other surface and secreted molecules from *N. gonorrhoeae* exposed dendritic cells in suppressing antigen induced T cell proliferation.

In transwell experiments, we showed that *N. gonorrhoeae* also suppressed T cell proliferation through DC secretion of soluble factors. IL-10 was upregulated and was required for full suppressive effect of *N. gonorrhoeae* in transwell-based experiments. Commensal bacteria-induced IL-10 secretion from antigen presenting cells is important in preventing uncontrolled colonic inflammation. Secreted IL-10 likely plays a role in preventing uncontrolled inflammation at other mucosal surfaces with high levels of commensal bacteria, like the vagina [39], [40]. Polymorphisms in the human *IL10* gene are associated with diminished T cell proliferation in response to chlamydial infection and with increased risk of tubal damage and infertility after chlamydial infection [41]. We showed that *N. gonorrhoeae* induces IL-10-mediated tolerance, paralleling this mechanism of immune suppression used by *Chlamydia trachomatis* [42]. IL-10 is elevated in cervical secretions of women infected with *C. trachomatis* and *N. gonorrhoeae*, suggesting this response occurs in clinical disease [43], [44]. Despite very different life cycles, these pathogens, both of which are highly adapted to the human lower genital tract, seem to have exploited similar host mechanisms in order to prevent effective adaptive immune responses.

PD-L1+, IL-10 expressing macrophages are responsible for initiating antigen tolerance in an autoimmune encephalitis model [45]. Both IL-10 and PD-L1 have been implicated in the development of a suppressive CD4 T cell population known as Treg. Interestingly, Treg cells are abundant in vaginal tissues of *N. gonorrhoeae*-infected mice [46], [47], [48]. Our data suggest that *N. gonorrhoeae* may prevent robust protective immune responses by programming host antigen presenting cells to induce tolerogenic responses, including Treg cells, directed towards gonococcal antigens. However, further research is needed to confirm that Treg polarization occurs in response to *N. gonorrhoeae* treated dendritic cells.

There are likely other host molecules involved in this pathogen-manipulated immunologic response. For example, we examined the expression of an array of genes during activation and antigen presentation by DCs using real time qRT-PCR (Figure S7 and Table 1). Culture with *N. gonorrhoeae* and ovalbumin stimulated greater than four-fold induction (compared to ovalbumin treatment alone) of 26 genes and resulted in a four-fold reduction in six genes. Seventeen of the 26 induced genes encode secreted cytokines or chemokines that are not known to suppress T cell proliferation. Steady-state mRNA encoding inhibin A was strongly upregulated (∼18-fold). Inhibin A (*Inhba*) induces tolerogenic signaling from DCs and is believed to be involved in immune tolerance during pregnancy [27]. Additional molecular tools including neutralizing antibodies, genetic inactivation, or siRNA-mediated silencing will be required to further investigate the role of inhibin A in *N. gonorrheoae*-induced immune tolerance. It is possible that non-biased gene expression profiling may reveal additional candidate genes whose expression is regulated in dendritic cells by exposure to *N. gonorrhoeae*.

**Table 1:** Fold change in genes over-expressed in BMDCs with *N. gonorrhoeae* (MOI = 1) plus OVA versus OVA only.

Gene	Relative expression (Ng+OVA:OVA Only treatment)
Cxcl1	855.4809
Il12a	187.5184
Cd40	161.802
Cxcl2	125.5174
Il12b	125.1483
Cxcl10	111.149
Il6	87.5084
Ccl17	26.7567
Ccl5	22.3534
Inhba	18.3513
Ifit3	16.9495
Ccl7	12.9143
Ccl3	11.6476
Ccl2	11.4902
Tnf	11.077
Erbb2	7.9394
Cd80	7.197
Cd1d2	7.0462
Cd86	7.0154
Ccl4	6.6678
Tlr1	5.6101
Cd4	5.2185
Ifng	5.0386
Ccl8	4.7898
Ccl19	4.5297
Fas	4.0752
Relb	3.9584
Cd209a	3.8636
Icam1	3.7087
Fcamr	3.5618
Il8ra	3.416
Ccl12	3.3347
Nfkb2	2.9508
Il2	2.9132
Nfkb1	2.9025
Ebi3	2.9012
Fcer1g	2.7914
Ccl20	2.726
Il23a	2.4116
Rela	2.3402
Tapbp	2.1733
B2m	2.1432
Cdkn1a	2.0629
Cxcl12	2.0003

Titration of *N. gonorrhoeae* demonstrated that at MOI<1, gonococci caused significant inhibition of dendritic cell-mediated antigen-induced T cell proliferation (Figure 2 F). This observation suggested that *N. gonorrhoeae* might exert an effect on dendritic cells in part through release of inhibitory factors into the culture medium. Prolific outer-membrane blebbing is a characteristic of pathogenic *Neisseria* species; however, the consequences of this process are not fully understood. Additionally, like *Bordetella pertussis*, *N. gonorrhoeae* releases high levels of anhydrous peptidoglycan monomers, also known as tracheal cytotoxin [49], [50], [51]. These released peptidoglycans are likely to be recognized by cellular peptidoglycan fragment sensing pattern recognition sensors, NOD1, NOD2, and TLR2. Recently, peptidoglycan-mediated activation of NOD2 and TLR2 signaling has been implicated in the upregulation of host PD-L1 and induction of immunologic tolerance [52]. Our data suggest that bleb-associated factors or peptidoglycan shedding may play an important role in manipulating the host immunologic response to *N. gonorrheoae* and possibly *N. meningitidis*.

The current findings indicate that *N. gonorrhoeae* likely suppresses protective host immune responses at the level of the antigen presenting cell, in addition to its direct effects on T and B lymphocytes, as previously reported [12], [13], [16], [19], [53]. We showed that *N. gonorrhoeae* suppresses dendritic cells' ability to induce CD4+ T cell proliferation in response to bacterially-expressed ovalbumin (Figure S2). Intracellular pathogens including *Salmonella enterica*, and *Mycobacterium bovus* and extracellular bacteria like *E. coli* have all previously been shown to induce specific proliferative responses when ovalbumin derived antigens are expressed by the bacteria [54], [55], [56]. Interestingly, *N. gonorrhoeae*-induced suppression can also be seen *in trans*, when antigens are co-delivered with the bacteria. This may have profound implications for STIs that are co-transmitted with *N. gonorrhoeae*, preventing the host's ability to mount immune responses to both *N. gonorrhoeae* and to other pathogens that are acquired at the same time. *N. gonorrhoeae* infection is associated with increased transmission of HIV, an effect likely mediated by the presence of increased viral burden in the semen of co-infected individuals as well as increased inflammation and HIV-susceptible cells at the sight of infection in HIV-negative individuals with gonorrhea [5]. It is certainly possible that the host immunologic response to HIV in the setting of *N. gonorrhoeae* infection may be hampered, leading to increased HIV acquisition rates in exposed individuals or impaired virologic control in those who are infected. Further studies into the mechanisms and gonococcal factors involved in this immuosuppressive effect may ultimately yield treatments or vaccine targets that will boost protective host responses to gonococcal antigens and possibly reduce the impact of *N. gonorrhoeae* infection on transmission of or host response to other sexually transmitted pathogens.

## Materials and Methods

### Ethics Statement

All protocols were conducted in accordance with National Institutes of Health guidelines for the care and use of laboratory animals and human subjects. The use of laboratory animals was approved by the Institutional Animal Care and Use Committee (IACUC) of the University of North Carolina at Chapel Hill (UNC IACUC protocol # 09-229.0). Human dendritic cells were generated from patients enrolled in a study approved by UNC IRB (Study #05-2860) after providing informed consent. The cells were provided as de-identified samples prior to utilization in the described studies. The use of the de-identified samples was reviewed by the UNC Office of Human Research Ethics, which determined that the study (Study #12-0024) does not constitute human subjects research as defined under federal regulations [45 CFR 46.102 (d or f) and 21 CFR 56.102(c)(e)(l)] and does not require further IRB approval.

### Preparation of N. gonorrhoeae

*N. gonorrhoeae* strain FA1090 was prepared as previously described [57]. Briefly, a predominantly Opa+ frozen stock of *N. gonorrhoeae* FA1090 was inoculated to GCB agar and grown overnight (16–18 hours) at 37°C and 5% CO_2_. The Opa protein expression was previously determined by whole cell immunoblotting 98 individual colonies and probing with a combination of five specific anti-Opa monoclonal antibodies, the greater than 80% of the colonies in the frozen population express at least one Opa with a predominance of OpaA, OpaD, and OpaI noted [57], [58]. Colonies were collected using a sterile cotton swab and inoculated to DMEM with 10% FCS. The bacterial density was estimated by measurement of OD_600_ and confirmed by plating serial dilutions. *N. gonorrhoeae* strains MS11 and F62 were prepared in a similar manner, though the specific Opa proteins expressed were not assessed for these two strains.

### Construction of OpaB-ovalbumin fusion protein expressing N. gonorrhoeae

A gene encoding a fusion protein in which peptides derived from *Gallus gallus* ovalbumin (257–264 and 323–339) were incorporated into the hyper-variable region, HV1, of OpaB was generated using a hybrid synthetic oligo/PCR approach (Figure S6). *OpaB* was amplified as two gene fragments from *N. gonorrhoeae* strain FA1090^opaA-K(B+)^, in which the expression of OpaB is phase locked on [59], and subcloned into the TOPO TA cloning vector (Invitrogen Corp., Carlsbad, CA). Nucleotides encoding an in-frame addition of ovalbumin amino acids 257–264 were incorporated into the oligonucleotide primer. These gene fragments were fused together at a synthetic SpeI site in pBluescript SK^+^ (Stratagene Cloning Systems). Synthetic DNA encoding OVA^323–339^ created from self-annealing oligos was inserted at the synthetic SpeI site creating a gene encoding the OpaB (OVA^(257–264;323–339)^) fusion protein. This gene was targeted to the *opaB* locus of gonococcal strain FA1090 using a two-step process. First, *N. gonorrhoeae* strain FA1090 was transformed with an *opaB* gene containing a *cat/rpsL* cassette within the HV-1 region and selected for chloramphenicol resistance and streptomycin sensitivity. Next, the *opaB::cat/rpsL* locus was replaced by homologous recombination with the *opaB::OVA* fusion gene and selected for streptomycin resistance and chloramphenicol sensitivity. OVA peptide expression in the gonococcus was confirmed by whole cell dot blot probed with a rabbit polyclonal anti-chicken ovalbumin sera (Bethyl Laboratories, Inc, Montgomery, TX), OpaB-specific monoclonal antibody H4, and porin P1B3-specific monoclonal antibody H5 (figure S2) [58].

### Culture of murine bone marrow-derived dendritic cells

BMDCs were prepared from 9–12 week old C57BL/6 mice (Jackson Laboratories, Bar Harbor, ME) as modified from a previous method [60]. In brief, femurs and tibiae were removed and left in 70% ethanol for 2–5 minutes and then washed with 1× PBS. Both ends of the bones were cut and bone marrow precursors were harvested by flushing with RPMI 1640 medium (Invitrogen Corp., Carlsbad, CA) supplemented with 10% FBS (Thermo Scientific, Logan, UT) using a syringe with 27 gauge needle (BD Biosciences, Franklin Lakes, NJ). Clusters within the marrow suspension were disintegrated by 21 gauge needle. Cells were centrifuged at 450× g for 8 minutes and cell pellets were then treated with 1X RBC lysis buffer [150 mM NH_4_Cl, 10 mM KHCO_3_, and 0.1 mM Na_2_EDTA (pH 7.4)] for 5 minutes at room temperature. After two additional washes, cell pellets were resuspended in 10% FBS RPMI 1640 medium containing GM-CSF (25 ng/mL; Peprotech, Rocky Hill, NJ) and IL-4 (10 ng/mL; Peprotech, Rocky Hill, NJ) and seeded into 6-well tissue culture plates at a density of 0.5×10^6^/mL with a total volume of 4 mL per well. Cultures were pulsed every 48 hours with fresh medium containing GM-CSF and IL-4. After 7 days in culture, immature DCs were harvested and used in T cell co-cultures as antigen presentation cells (APCs).

### Infection and stimulation of BMDC

BMDCs were washed and resuspended in antibiotic-free medium at a density of 1×10^6^/mL. Cells were infected with *N. gonorrhoeae* strain FA1090 with the indicated multiplicity of infection (MOI) in the absence or presence of soluble Ovalbumin (OVA; 100 µg/mL; Sigma-Aldrich, St. Louis, MO) [57]. BMDCs stimulated with OVA or medium only served as controls. Four hours post-infection or treatment, BMDC cultures were supplemented with 50 µg/mL Gentamicin (Invitrogen Corp., Carlsbad, CA) to kill extracellular bacteria. Cultures were returned to 37°C, 5% CO_2_ humidified incubators and harvested 24 hours post infection/treatment for co-culture with T cells or down-stream assays.

To determine the survival of *N. gonorrhoeae* within murine BMDC, Murine BMDCs were inoculated with either MOI 1 or MOI 10 *N. gonorrhoeae* and incubated at 37°C 5% CO_2_ in RPMI 1640 supplemented with 10% heat-inactivated fetal bovine serum in 5 mL polystyrene tubes. After 4 h, cells were spun at 2500 rpm and washed 2× with 1 mL fresh medium (t = 0 h). Gentamicin was added to the cells to a concentration of 50 µg/mL gentamicin for 1 hour. Subsequently, cells were washed 2× with medium (t = 1 h) and allowed to grow for an additional 19 hours (t = 20 h) at 37°C 5% CO_2_. At each time point (t = 0 h, 1 h, 20 h) cell-associated bacteria were determined by adding saponin to 1% for 10 minutes followed by plating serial dilutions on GCB Agar plates in triplicate. After a 48 h growth period, colonies were counted using Synbiosis aCOLyte colony counter.

### Measurement of cytokines/chemokines, TLR2 activation and caspase-3 activation

Mouse KC, TNF-α, MIP-1β, RANTES, IL-2 and IL-10 levels in cell culture supernatants were determined by multiplex bead-based assays using Bio-Plex Pro™ Mouse Cytokine assays (BioRad, Hercules, CA) according to the manufacturer's protocol. Bead assays were quantified on the Bio-Plex protein array reader (BioRad, Hercules, CA) in the Duke Human Vaccine Institute Immune Reconstitution and Biomarker Facility.

### DQ-OVA endocytosis assay

DQ-OVA (Invitrogen Corp., Carlsbad, CA) is a self-quenched conjugate of ovalbumin that exhibits bright green fluorescence upon proteolytic degradation and can be measured by flow cytometry. BMDCs were resuspended in antibiotic-free medium at a density of 1×10^6^/mL and pulsed with DQ-OVA (10 µg/mL) with or without *N. gonorrhoeae* (MOI = 1). BMDCs were incubated at 37°C for 1, 4 or 24 hours. Gentamicin was added to kill extracellular *N. gonorrhoeae* at 1 or 4 hours after infection. BMDCs were collected at various time points, washed three times with cold 1× PBS and re-suspended in FACS buffer (1× PBS, 1% BSA, 0.1% NaN_3_). Cells were evaluated immediately via flow cytometry for DQ-OVA uptake and processing. Controls include cells with DQ-OVA at 4°C and cells incubated at 37°C without DQ-OVA.

### BMDC-T Cell co-culture/proliferation assay

Spleen and lymph nodes were excised from OT-II mice (obtained from Jackson Laboratories, Bar Harbor, ME) and single-cell suspensions were prepared by dissociating cells through 70 µm cell strainers (BD, Bedford, MA) and removing red cells with 1X RBC lysis buffer. Splenocytes/LN cells were first separated by lymphocyte separation medium (LSM, Accurate Chemical & Scientific Corporation, Westbury, NY) and then passed over T cell enrichment columns (R&D Systems, Minneapolis, MN). For BMDC/T co-culture experiments, enriched T cells (0.5×10^6^/mL) were labeled with carboxy fluoroscein succinimidyl ester (CFSE; Molecular Probes, Eugene, OR) and mixed with 0.5×10^5^/mL of pretreated BMDCs. Cells were cultured in RPMI 1640 medium containing 10% FBS in a total volume of 1 mL per well in 48-well tissue culture plates or in tissue culture plates containing transwell (0.4 µm) inserts (Costar, Corning, NY). After 7 days, BMDC/T cells were harvested and T cell proliferation was measured by flow cytometric analysis of CFSE dilution.

### Generation of human dendritic cells and mixed lymphocyte reaction (MLR) assay

Primary human dendritic cells were generated by culture of CD34+ cells from peripheral blood in the presence of Stem Cell Factor (SCF 50 ng/mL) Flt3L (100 ng/mL), GM-CSF(800 U/mL) and IL-4(500 U/mL) in AIM V medium with 10% human AB serum for 14 days. Human peripheral blood mononuclear cells were depleted of antigen-presenting cells by adherence to T75 tissue culture flask supplied with 10% AB serum AIM V medium without agitation. Two hours later, non-adherent (NAD) cells were collected and incubated into another T75 tissue culture flask for another 2 hours incubation. The NAD cells were collected and pooled from 5 donors and used as responder cells and labeled with CFSE. These CFSE-labeled lymphocytes were co-cultured with medium or *N. gonorrhoeae* exposed human DCs (MOI 1, 10) in 96-well U-bottom plate. CFSE-labeled NAD cells (responder cells) were plated at 1×10^5^ cells per well in a volume of 200 µL and co-cultured with human DCs (stimulator cells) at a ratio of 3∶1, 10∶1 and 30∶1. NAD cells alone were used as negative control. After co-cultured for 3 and 7 days, mixed lymphocyte cultures were harvested and stained with CD4-PE-Cy5, CD4^+^ T cell proliferation was then measured by flow cytometric analysis of CFSE dilution.

### Immunophenotyping and flow cytometry

Polychromatic immunophenotyping was performed using peridinin chlorophyll protein conjugated to the cyanine dye 5.5 (PerCP-Cy5.5), phycoerythrin conjugated to the cyanine dye 5 (PE-Cy5), phycoerythrin conjugated to the cyanine dye 7 (PE-Cy7), allophycocyanin (APC), allophycocyanin conjugated to the cyanine dye 7 (APC-Cy7) and Alexa Fluor 700 as fluorescent dyes. Directly-conjugated anti-mouse monoclonal antibodies were used against: CD3-APC, CD3-APC-Cy7, CD3-PE-Cy7, CD4-APC-Cy7, CD8-PE-Cy7, CD80-PE, CD86-PE-Cy5, CD45R/B220-PE-Cy7, CD11c-APC-Cy7, CD273-APC and CD274-PE (BD Biosciences, San Jose, CA and eBioscience, San Diego, CA). Biotin anti-mouse V beta 5.1/5.2, Biotin anti-mouse I-A [b] and APC Streptavidin were also used (BD Biosciences, San Jose, CA). Cell viability was assessed with AnexinV-PE and 7AAD (BD Biosciences, San Jose, CA), and Caspase-3 was detected with FLICA and propidium iodide (PI) (Immunochemistry Technologies, Bloomington, MN).

Saturating amounts of antibody were used to stain approximately 1×10^6^ cells in FACS Buffer (1× PBS, 1% BSA, 0.1% NaN_3_) (final volume of 100 µL) at 25°C for 1 hour. All samples were washed with 3 mL FACS Buffer and resuspended in 200 µL of FACS Buffer with 0.4% (w/v) paraformaldehyde. Stained samples were analyzed either on a BD-FACS Canto or a BD LSRII-SOS (BD Biosciences, Palo Alto, CA) in the Duke Human Vaccine Institute Flow Cytometry Facility. For each sample, forward and side angle light scatter profiles were used to acquire 10,000–100,000 events. Data were saved as FCS 3.0 and analyzed with FlowJo software (Tree Star, Inc. Ashland, OR). When necessary, the gating strategies are introduced in the respective figures as a representative scatter plot. In some experiments, the comparison of a single fluorescent parameter between two experimental groups is performed using overlayed histograms in which the Y values are normalized to the peak value in that sample in order to facilitate the comparison of the distribution of fluorescence in each population. In these cases, the Y-axis is labeled as % Max.

### Real-time reverse-transcription PCR arrays

Quantitative real-time RT-PCR was used to profile expression of a panel of genes involved in antigen presentation (Table 1) and the additional genes in BMDC cultures. Total RNA was extracted from cell pellets using the RNeasy Mini Kit (Qiagen, Valencia, CA) per manufacturer's instructions, and quantified on a Nanodrop spectrophotometer (Thermo Scientific, Wilmington, DE). Isolated RNA (500 ng) was reverse transcribed, and real-time PCR performed, following the provided protocols with RT^2^ First-Strand cDNA Synthesis Kit and Mouse Dendritic and Antigen Presenting Cell or Custom Mouse PCR Array, respectively (SA Biosciences, Frederick, MD). Relative gene expression was quantified using the comparative C_T_ method (ΔΔC_T_) via the PCR Array Data Analysis Web Portal at http://www.sabiosciences.com/pcrarraydataanalysis.php.

## Supporting Information

Figure S1 ***N. gonorrhoeae*** **does not survive intracellularly in murine BMDC.** Murine BMDCs were incubated with the indicated dose (MOI 1 & MOI 10) of *N. gonorrhoeae* for 4 hours. Extracellular bacteria were removed from the culture by washing, followed by 1-hour treatment with gentamicin and subsequent culture of the BMDC for an additional 19 hours. The quantity of BMDC-associated *N. gonorrhoeae* was assessed by lysing washed BMDC with saponin and plating serial dilutions at the following time points: after 4 h incubation with mouse BMDCs (0 h); after an additional 1 h incubation with gentamicin (1 h); and following an additional 19 h of growth (20 h). Mean colony forming units (cfu) +/− S.E.M. from triplicate plates are plotted. Asterix (*) indicates below the detectable limit (6 cfu).
(TIF)

Figure S2 **OVA-expressing** ***N. gonorrhoeae*** **inhibits BMDCs antigen-induced T cell proliferation.** **A**) The predicted membrane topology of the OpaB (OVA^(257–264;323–339)^) fusion protein is shown in two dimensions. The hypervariable (HV) regions are indicated by hashed lines and the insertion of OVA^(257–264;323–339)^ into hyper variable region-1 (HV1) is indicated. **B**) The indicated strains of *N. gonorrhoeae* strains were grown for 18 hours, harvested, and resuspended. The resuspended bacteria (100 µL, 0.2 OD600) or isolated OVA (1.0 µg) were spotted to nitrocellulose and probed with the indicated antibodies as described in the materials and methods. **C–G**) BMDCs were exposed to OVA-expressing *N. gonorrhoeae* (MOI = 1) with or without OVA for 24 hours and then co-cultured with CFSE-loaded OT-II T cells for 7 days. OT-II T cell proliferation to OVA was assessed by flow cytometric analysis (CFSE dilution). **C**) Representative gating strategy of CD4+ Vβ5+ OT-II T cells. Representative OT-II T cell proliferation profile following co-culture with BMDCs treated with **D**) medium only, **E**) OVA (100 µg/mL), **F**) OVA-expressing *N. gonorrhoeae* (MOI = 1).
(TIFF)

Figure S3 ***N. gonorrhoeae*** **does not impact OVA induced IL-2 production in T-cell/BMDC co-culture.** BMDCs were exposed to *N. gonorrhoeae* at MOI of 1 with or without OVA for 24 hours and then co-cultured with OT-II T cells for seven days as described in Figure 2. Secreted IL-2 levels in culture supernatant were measured using a multiplex bead assay in seven-day DC-T cell co-culture supernatant N = 4, ND = Not Detectable).
(TIF)

Figure S4 ***N. gonorrhoeae*** **-treated BMDCs from** ***Il10^−/−^*** **mice demonstrate similar inhibition on T cell proliferation as seen with WT BMDCs.** BMDCs were exposed to *N. gonorrhoeae* at different MOIs with or without OVA for 24 hours and then co-cultured with CFSE-loaded OT-II T cells for seven days. T cell proliferation to OVA was assessed by flow cytometric analysis. Percent proliferation of T cells normalized to OVA-DC-induced T cell proliferation. Data are mean ± standard deviation (N = 3).
(TIF)

Figure S5 **CD4+ T cell apoptosis is unchanged by BMDC exposure to** ***N. gonorrhoeae*** **in the absence of antigen.** Caspase 3&7 activity (FLICA) form CD4+ Vβ5+ OT-II T cells following co-culture with medium or *N. gonorrhoeae* (MOI = 1) pulsed BMDCs. Percentage of apoptotic CD4+ Vβ5+ OT-II T cells following co-cultured with BMDCs for 24 hours. Data are mean ± standard deviation (N = 4 replicates).
(TIFF)

Figure S6 **Construction of OpaB(OVA^(257–264;323–339)^) -expressing** ***N. gonorrhoeae*** **FA1090 strain.** *N. gonorrhoeae OpaB* containing intermediate plasmid constructs used to generate an OpaB (OVA^(257–264;323–339)^)-expressing *N. gonorrhoeae* strain are shown (designated pJAD). Oligonucleotides used to amplify segments of OpaB or insert sequences encoding amino acids 323 to 339 of *G. gallus* ovalbumin are designated OVA-1 to OVA-6.
(TIF)

Figure S7 **Expression of inflammatory genes was upregulated in BMDCs 24 hours post** ***N. gonorrhoeae*** **exposure.** Representative scatter plots of gene expression (qRT-PCR arrays) from **A**) medium only versus OVA-pulsed BMDCs and **B**) OVA-pulsed BMDCs versus *N. gonorrhoeae* (MOI = 1) with OVA, N = 3.
(TIFF)

## References

[1] Tapsall JW (2005). Antibiotic resistance in Neisseria gonorrhoeae.. Clin Infect Dis.

[2] Handsfield HH, Lipman TO, Harnisch JP, Tronca E, Holmes KK (1974). Asymptomatic gonorrhea in men. Diagnosis, natural course, prevalence and significance.. The New England journal of medicine.

[3] Korenromp EL, Sudaryo MK, de Vlas SJ, Gray RH, Sewankambo NK (2002). What proportion of episodes of gonorrhoea and chlamydia becomes symptomatic?. International journal of STD & AIDS.

[4] Cohen MS, Hoffman IF, Royce RA, Kazembe P, Dyer JR (1997). Reduction of concentration of HIV-1 in semen after treatment of urethritis: implications for prevention of sexual transmission of HIV-1. AIDSCAP Malawi Research Group.. Lancet.

[5] Ghys PD, Fransen K, Diallo MO, Ettiegne-Traore V, Coulibaly IM (1997). The associations between cervicovaginal HIV shedding, sexually transmitted diseases and immunosuppression in female sex workers in Abidjan, Cote d'Ivoire.. Aids.

[6] Fox KK, Thomas JC, Weiner DH, Davis RH, Sparling PF (1999). Longitudinal evaluation of serovar-specific immunity to Neisseria gonorrhoeae.. Am J Epidemiol.

[7] Cohen IR (1967). Natural and immune human antibodies reactive with antigens of virulent Neisseria gonorrhoeae: immunoglobulins G, M, And A. Journal of bacteriology.

[8] Tapchaisri P, Sirisinha S (1976). Serum and secretory antibody responses to Neisseria gonorrhoeae in patients with gonococcal infections.. The British journal of venereal diseases.

[9] Massari P, Henneke P, Ho Y, Latz E, Golenbock DT (2002). Cutting edge: Immune stimulation by neisserial porins is toll-like receptor 2 and MyD88 dependent.. Journal of immunology.

[10] Pridmore AC, Jarvis GA, John CM, Jack DL, Dower SK (2003). Activation of toll-like receptor 2 (TLR2) and TLR4/MD2 by Neisseria is independent of capsule and lipooligosaccharide (LOS) sialylation but varies widely among LOS from different strains.. Infection and Immunity.

[11] Steeghs L, van Vliet SJ, Uronen-Hansson H, van Mourik A, Engering A (2006). Neisseria meningitidis expressing lgtB lipopolysaccharide targets DC-SIGN and modulates dendritic cell function.. Cellular microbiology.

[12] Pantelic M, Kim YJ, Bolland S, Chen I, Shively J (2005). Neisseria gonorrhoeae kills carcinoembryonic antigen-related cellular adhesion molecule 1 (CD66a)-expressing human B cells and inhibits antibody production.. Infect Immun.

[13] Boulton IC, Gray-Owen SD (2002). Neisserial binding to CEACAM1 arrests the activation and proliferation of CD4+ T lymphocytes.. Nat Immunol.

[14] Chen T, Grunert F, Medina-Marino A, Gotschlich EC (1997). Several carcinoembryonic antigens (CD66) serve as receptors for gonococcal opacity proteins.. The Journal of experimental medicine.

[15] Chen T, Gotschlich EC (1996). CGM1a antigen of neutrophils, a receptor of gonococcal opacity proteins.. Proceedings of the National Academy of Sciences of the United States of America.

[16] Lee HS, Ostrowski MA, Gray-Owen SD (2008). CEACAM1 dynamics during neisseria gonorrhoeae suppression of CD4+ T lymphocyte activation.. Journal of immunology.

[17] McCaw SE, Liao EH, Gray-Owen SD (2004). Engulfment of Neisseria gonorrhoeae: revealing distinct processes of bacterial entry by individual carcinoembryonic antigen-related cellular adhesion molecule family receptors.. Infect Immun.

[18] Youssef AR, van der Flier M, Estevao S, Hartwig NG, van der Ley P (2009). Opa+ and Opa− isolates of Neisseria meningitidis and Neisseria gonorrhoeae induce sustained proliferative responses in human CD4+ T cells.. Infection and Immunity.

[19] Plant LJ, Jonsson AB (2006). Type IV pili of Neisseria gonorrhoeae influence the activation of human CD4+ T cells.. Infect Immun.

[20] Feinen B, Jerse AE, Gaffen SL, Russell MW (2010). Critical role of Th17 responses in a murine model of Neisseria gonorrhoeae genital infection.. Mucosal immunology.

[21] Gagliardi MC, Starnino S, Teloni R, Mariotti S, Dal Conte I (2011). Circulating levels of interleukin-17A and interleukin-23 are increased in patients with gonococcal infection.. FEMS immunology and medical microbiology.

[22] Criss AK, Katz BZ, Seifert HS (2009). Resistance of Neisseria gonorrhoeae to non-oxidative killing by adherent human polymorphonuclear leucocytes.. Cellular microbiology.

[23] Witt K, Veale DR, Smith H (1976). Resistance of Neisseria gonorrhoeae to ingestion and digestion by phagocytes of human buffy coat.. Journal of medical microbiology.

[24] Liu Y, Russell MW (2011). Diversion of the Immune Response to Neisseria gonorrhoeae from Th17 to Th1/Th2 by Treatment with Anti-Transforming Growth Factor {beta} Antibody Generates Immunological Memory and Protective Immunity.. mBio 2.

[25] Barnden MJ, Allison J, Heath WR, Carbone FR (1998). Defective TCR expression in transgenic mice constructed using cDNA-based alpha- and beta-chain genes under the control of heterologous regulatory elements.. Immunology and cell biology.

[26] Malorny B, Morelli G, Kusecek B, Kolberg J, Achtman M (1998). Sequence diversity, predicted two-dimensional protein structure, and epitope mapping of neisserial Opa proteins.. Journal of bacteriology.

[27] Mondino A, Jenkins MK (1994). Surface proteins involved in T cell costimulation.. Journal of leukocyte biology.

[28] Manicassamy S, Ravindran R, Deng J, Oluoch H, Denning TL (2009). Toll-like receptor 2-dependent induction of vitamin A-metabolizing enzymes in dendritic cells promotes T regulatory responses and inhibits autoimmunity.. Nature medicine.

[29] Zhang Y, Chung Y, Bishop C, Daugherty B, Chute H (2006). Regulation of T cell activation and tolerance by PD-L2.. Proceedings of the National Academy of Sciences of the United States of America.

[30] Dong H, Strome SE, Salomao DR, Tamura H, Hirano F (2002). Tumor-associated B7-H1 promotes T-cell apoptosis: a potential mechanism of immune evasion.. Nature medicine.

[31] Chang CC, Ciubotariu R, Manavalan JS, Yuan J, Colovai AI (2002). Tolerization of dendritic cells by T(S) cells: the crucial role of inhibitory receptors ILT3 and ILT4.. Nature immunology.

[32] Vlad G, Chang CC, Colovai AI, Vasilescu ER, Cortesini R (2010). Membrane and soluble ILT3 are critical to the generation of T suppressor cells and induction of immunological tolerance.. International reviews of immunology.

[33] Baba N, Samson S, Bourdet-Sicard R, Rubio M, Sarfati M (2008). Commensal bacteria trigger a full dendritic cell maturation program that promotes the expansion of non-Tr1 suppressor T cells.. Journal of leukocyte biology.

[34] Marri PR, Paniscus M, Weyand NJ, Rendon MA, Calton CM (2010). Genome sequencing reveals widespread virulence gene exchange among human Neisseria species.. PLoS One.

[35] Detels R, Green AM, Klausner JD, Katzenstein D, Gaydos C (2011). The incidence and correlates of symptomatic and asymptomatic Chlamydia trachomatis and Neisseria gonorrhoeae infections in selected populations in five countries.. Sexually transmitted diseases.

[36] Guleria I, Khosroshahi A, Ansari MJ, Habicht A, Azuma M (2005). A critical role for the programmed death ligand 1 in fetomaternal tolerance.. The Journal of experimental medicine.

[37] Jounai K, Ikado K, Sugimura T, Ano Y, Braun J (2012). Spherical lactic acid bacteria activate plasmacytoid dendritic cells immunomodulatory function via TLR9-dependent crosstalk with myeloid dendritic cells.. PLoS One.

[38] Peng B, Lu C, Tang L, Yeh IT, He Z (2011). Enhanced upper genital tract pathologies by blocking Tim-3 and PD-L1 signaling pathways in mice intravaginally infected with Chlamydia muridarum.. BMC infectious diseases.

[39] Albright CA, Sartor RB, Tonkonogy SL (2009). Endogenous antigen presenting cell-derived IL-10 inhibits T lymphocyte responses to commensal enteric bacteria.. Immunology letters.

[40] Larsen JM, Steen-Jensen DB, Laursen JM, Sondergaard JN, Musavian HS (2012). Divergent pro-inflammatory profile of human dendritic cells in response to commensal and pathogenic bacteria associated with the airway microbiota.. PLoS One.

[41] Ohman H, Tiitinen A, Halttunen M, Lehtinen M, Paavonen J (2009). Cytokine polymorphisms and severity of tubal damage in women with Chlamydia-associated infertility.. The Journal of Infectious Diseases.

[42] Marks E, Tam MA, Lycke NY (2010). The female lower genital tract is a privileged compartment with IL-10 producing dendritic cells and poor Th1 immunity following Chlamydia trachomatis infection.. PLoS pathogens.

[43] Cohen CR, Plummer FA, Mugo N, Maclean I, Shen C (1999). Increased interleukin-10 in the the endocervical secretions of women with non-ulcerative sexually transmitted diseases: a mechanism for enhanced HIV-1 transmission?. Aids.

[44] Geisler WM, Wang C, Tang J, Wilson CM, Crowley-Nowick PA (2008). Immunogenetic correlates of Neisseria gonorrhoeae infection in adolescents.. Sexually transmitted diseases.

[45] Getts DR, Turley DM, Smith CE, Harp CT, McCarthy D (2011). Tolerance induced by apoptotic antigen-coupled leukocytes is induced by PD-L1+ and IL-10−producing splenic macrophages and maintained by T regulatory cells.. Journal of immunology.

[46] Bellinghausen I, Konig B, Bottcher I, Knop J, Saloga J (2006). Inhibition of human allergic T-helper type 2 immune responses by induced regulatory T cells requires the combination of interleukin-10-treated dendritic cells and transforming growth factor-beta for their induction.. Clinical and experimental allergy : journal of the British Society for Allergy and Clinical Immunology.

[47] Wan YY, Flavell RA (2008). TGF-beta and regulatory T cell in immunity and autoimmunity.. Journal of clinical immunology.

[48] Strauss L, Bergmann C, Szczepanski M, Gooding W, Johnson JT (2007). A unique subset of CD4+CD25highFoxp3+ T cells secreting interleukin-10 and transforming growth factor-beta1 mediates suppression in the tumor microenvironment.. Clinical cancer research : an official journal of the American Association for Cancer Research.

[49] Cookson BT, Tyler AN, Goldman WE (1989). Primary structure of the peptidoglycan-derived tracheal cytotoxin of Bordetella pertussis.. Biochemistry.

[50] Goldman WE, Herwaldt LA (1985). Bordetella pertussis tracheal cytotoxin.. Developments in biological standardization.

[51] Sinha RK, Rosenthal RS (1980). Release of soluble peptidoglycan from growing conococci: demonstration of anhydro-muramyl-containing fragments.. Infection and Immunity.

[52] Hewitt RE, Pele LC, Tremelling M, Metz A, Parkes M (2012). Immuno-inhibitory PD-L1 can be induced by a Peptidoglycan/NOD2 mediated pathway in primary monocytic cells and is deficient in Crohn's patients with homozygous NOD2 mutations.. Clinical immunology.

[53] van Vliet SJ, Steeghs L, Bruijns SC, Vaezirad MM, Snijders Blok C (2009). Variation of Neisseria gonorrhoeae lipooligosaccharide directs dendritic cell-induced T helper responses.. PLoS pathogens.

[54] Morel C, Badell E, Abadie V, Robledo M, Setterblad N (2008). Mycobacterium bovis BCG-infected neutrophils and dendritic cells cooperate to induce specific T cell responses in humans and mice.. European journal of immunology.

[55] Svensson M, Johansson C, Wick MJ (2000). Salmonella enterica serovar typhimurium-induced maturation of bone marrow-derived dendritic cells.. Infection and Immunity.

[56] Serre K, Mohr E, Toellner KM, Cunningham AF, Granjeaud S (2008). Molecular differences between the divergent responses of ovalbumin-specific CD4 T cells to alum-precipitated ovalbumin compared to ovalbumin expressed by Salmonella.. Molecular immunology.

[57] Duncan JA, Gao X, Huang MT, O'Connor BP, Thomas CE (2009). Neisseria gonorrhoeae activates the proteinase cathepsin B to mediate the signaling activities of the NLRP3 and ASC-containing inflammasome.. J Immunol.

[58] Jerse AE, Cohen MS, Drown PM, Whicker LG, Isbey SF (1994). Multiple gonococcal opacity proteins are expressed during experimental urethral infection in the male.. J Exp Med.

[59] Cole JG, Fulcher NB, Jerse AE (2010). Opacity proteins increase Neisseria gonorrhoeae fitness in the female genital tract due to a factor under ovarian control.. Infection and Immunity.

[60] Fields RC, Osterholzer JJ, Fuller JA, Thomas EK, Geraghty PJ (1998). Comparative analysis of murine dendritic cells derived from spleen and bone marrow.. Journal of immunotherapy.

