# Supplementary figures and images for: Neisseria gonorrhoeae Suppresses Dendritic Cell-Induced, Antigen-Dependent CD4 T Cell Proliferation
Source: PLoS One. 2012 Jul 23;7(7):e41260. doi: 10.1371/journal.pone.0041260 (PMC3402525; doi:10.1371/journal.pone.0041260)

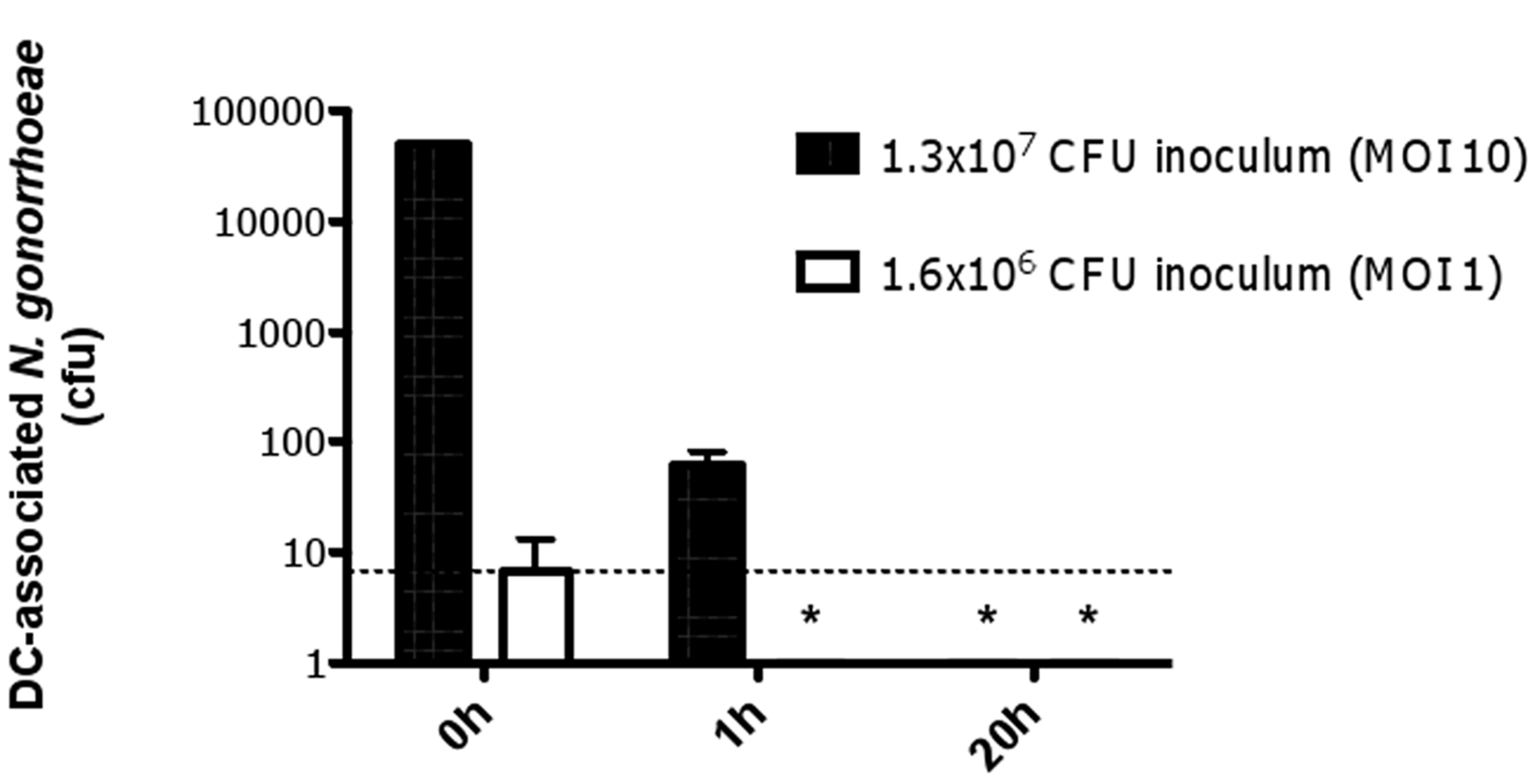

Supplement: Figure S1 — N. gonorrhoeae does not survive intracellularly in murine BMDC. Murine BMDCs were incubated with the indicated dose (MOI 1 & MOI 10) of N. gonorrhoeae for 4 hours. Extracellular bacteria were removed from the culture by washing, followed by 1-hour treatment with gentamicin and subsequent culture of the BMDC for an additional 19 hours. The quantity of BMDC-associated N. gonorrhoeae was assessed by lysing washed BMDC with saponin and plating serial dilutions at the following time points: after 4 h incubation with mouse BMDCs (0 h); after an additional 1 h incubation with gentamicin (1 h); and following an additional 19 h of growth (20 h). Mean colony forming units (cfu) +/− S.E.M. from triplicate plates are plotted. Asterix (*) indicates below the detectable limit (6 cfu). (TIF) [file pone.0041260.s001.tif]

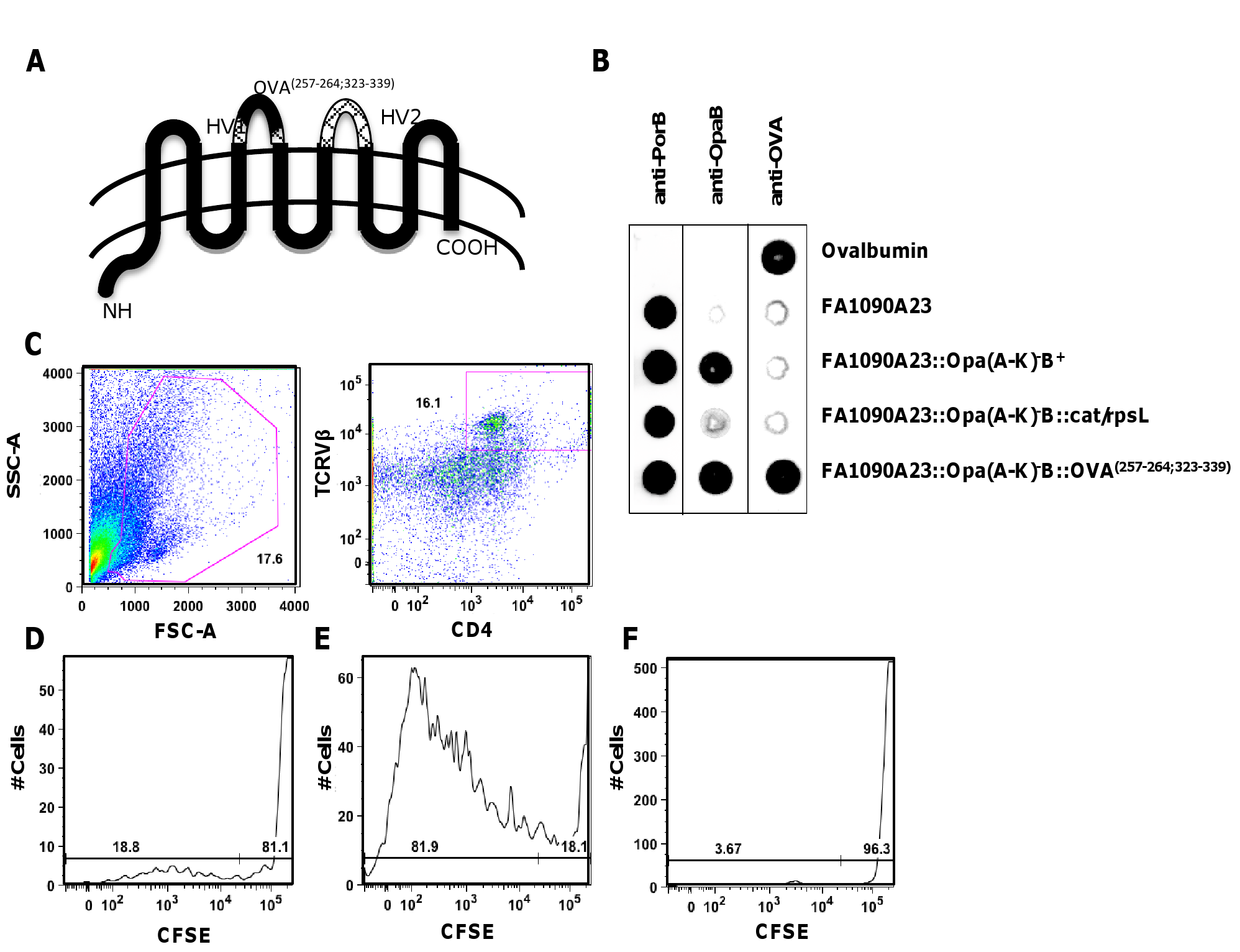

Supplement: Figure S2 — OVA-expressing N. gonorrhoeae inhibits BMDCs antigen-induced T cell proliferation. A) The predicted membrane topology of the OpaB (OVA(257–264;323–339)) fusion protein is shown in two dimensions. The hypervariable (HV) regions are indicated by hashed lines and the insertion of OVA(257–264;323–339) into hyper variable region-1 (HV1) is indicated. B) The indicated strains of N. gonorrhoeae strains were grown for 18 hours, harvested, and resuspended. The resuspended bacteria (100 µL, 0.2 OD600) or isolated OVA (1.0 µg) were spotted to nitrocellulose and probed with the indicated antibodies as described in the materials and methods. C–G) BMDCs were exposed to OVA-expressing N. gonorrhoeae (MOI = 1) with or without OVA for 24 hours and then co-cultured with CFSE-loaded OT-II T cells for 7 days. OT-II T cell proliferation to OVA was assessed by flow cytometric analysis (CFSE dilution). C) Representative gating strategy of CD4+ Vβ5+ OT-II T cells. Representative OT-II T cell proliferation profile following co-culture with BMDCs treated with D) medium only, E) OVA (100 µg/mL), F) OVA-expressing N. gonorrhoeae (MOI = 1). (TIFF) [file pone.0041260.s002.tiff]

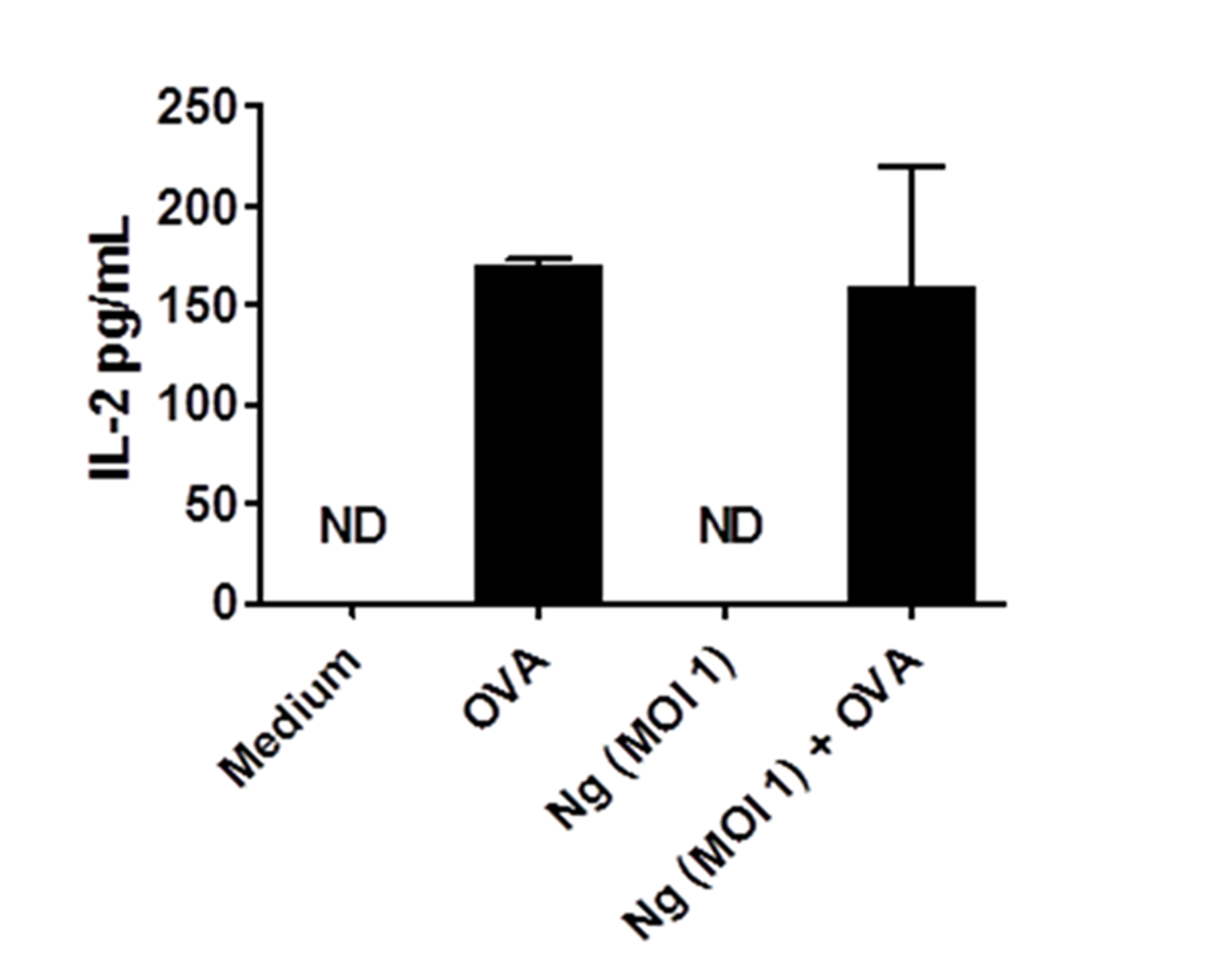

Supplement: Figure S3 — N. gonorrhoeae does not impact OVA induced IL-2 production in T-cell/BMDC co-culture. BMDCs were exposed to N. gonorrhoeae at MOI of 1 with or without OVA for 24 hours and then co-cultured with OT-II T cells for seven days as described in Figure 2. Secreted IL-2 levels in culture supernatant were measured using a multiplex bead assay in seven-day DC-T cell co-culture supernatant N = 4, ND = Not Detectable). (TIF) [file pone.0041260.s003.tif]

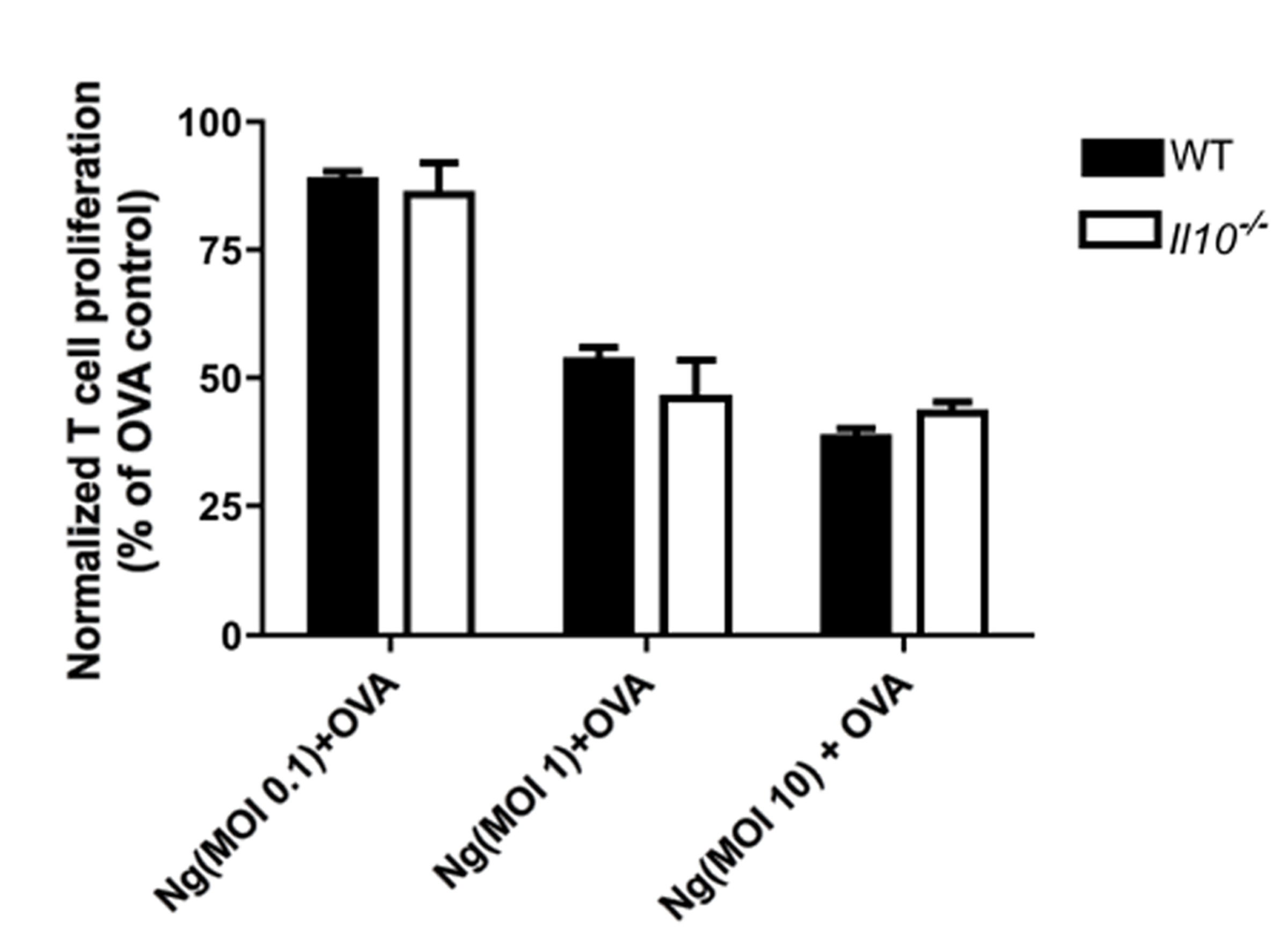

Supplement: Figure S4 — N. gonorrhoeae -treated BMDCs from Il10−/− mice demonstrate similar inhibition on T cell proliferation as seen with WT BMDCs. BMDCs were exposed to N. gonorrhoeae at different MOIs with or without OVA for 24 hours and then co-cultured with CFSE-loaded OT-II T cells for seven days. T cell proliferation to OVA was assessed by flow cytometric analysis. Percent proliferation of T cells normalized to OVA-DC-induced T cell proliferation. Data are mean ± standard deviation (N = 3). (TIF) [file pone.0041260.s004.tif]

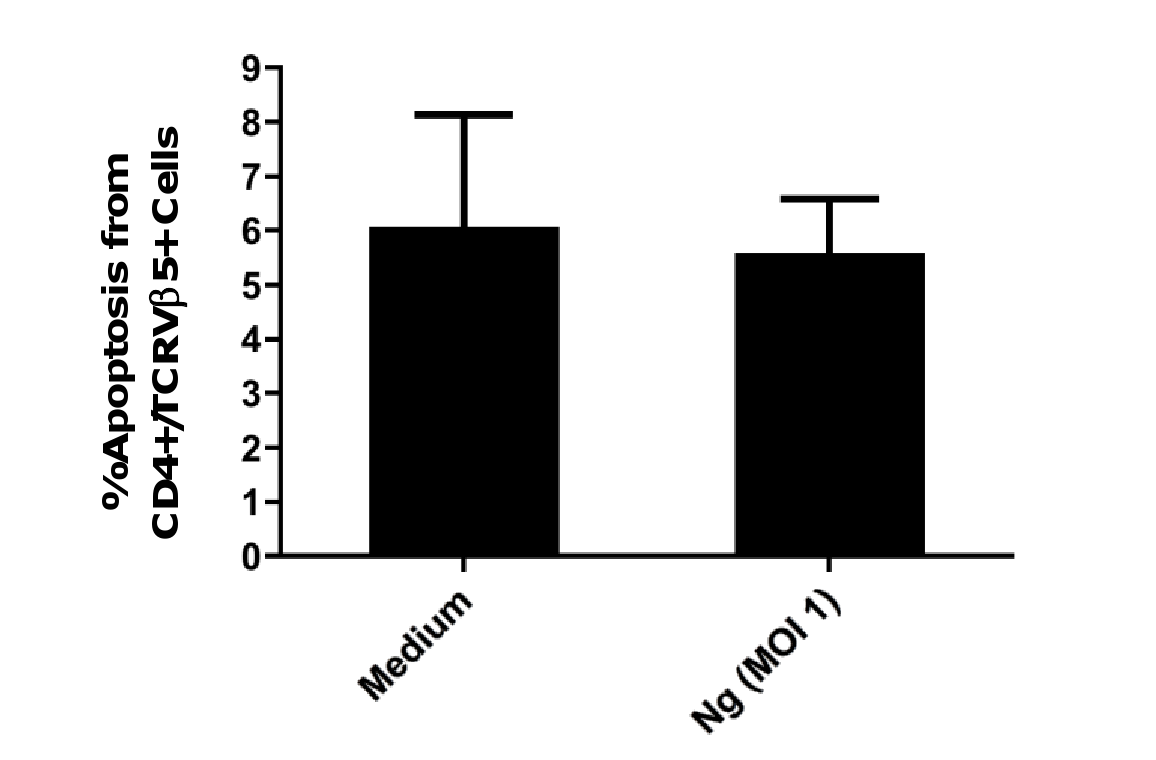

Supplement: Figure S5 — CD4+ T cell apoptosis is unchanged by BMDC exposure to N. gonorrhoeae in the absence of antigen. Caspase 3&7 activity (FLICA) form CD4+ Vβ5+ OT-II T cells following co-culture with medium or N. gonorrhoeae (MOI = 1) pulsed BMDCs. Percentage of apoptotic CD4+ Vβ5+ OT-II T cells following co-cultured with BMDCs for 24 hours. Data are mean ± standard deviation (N = 4 replicates). (TIFF) [file pone.0041260.s005.tiff]

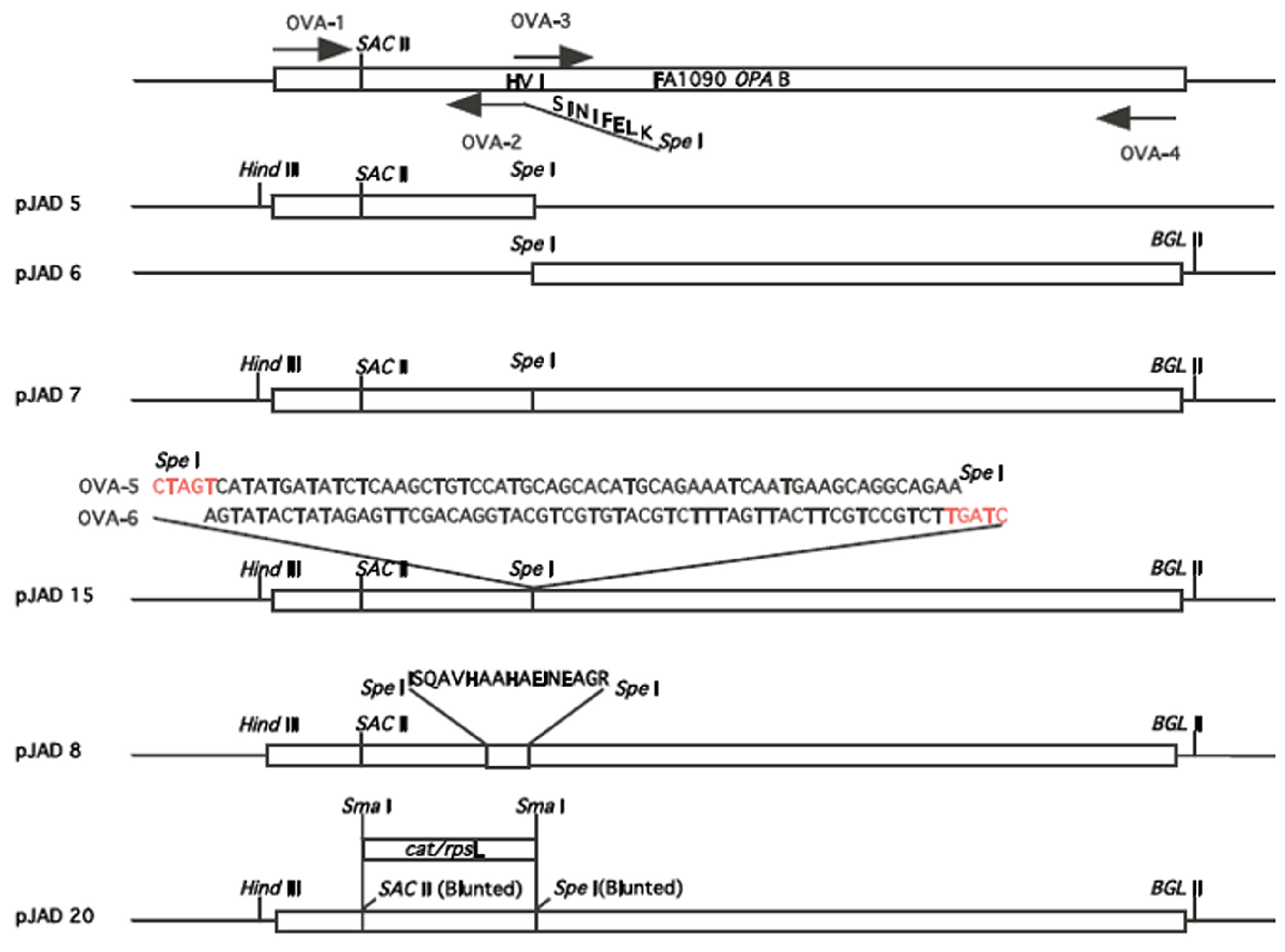

Supplement: Figure S6 — Construction of OpaB(OVA(257–264;323–339)) -expressing N. gonorrhoeae FA1090 strain. N. gonorrhoeae OpaB containing intermediate plasmid constructs used to generate an OpaB (OVA(257–264;323–339))-expressing N. gonorrhoeae strain are shown (designated pJAD). Oligonucleotides used to amplify segments of OpaB or insert sequences encoding amino acids 323 to 339 of G. gallus ovalbumin are designated OVA-1 to OVA-6. (TIF) [file pone.0041260.s006.tif]

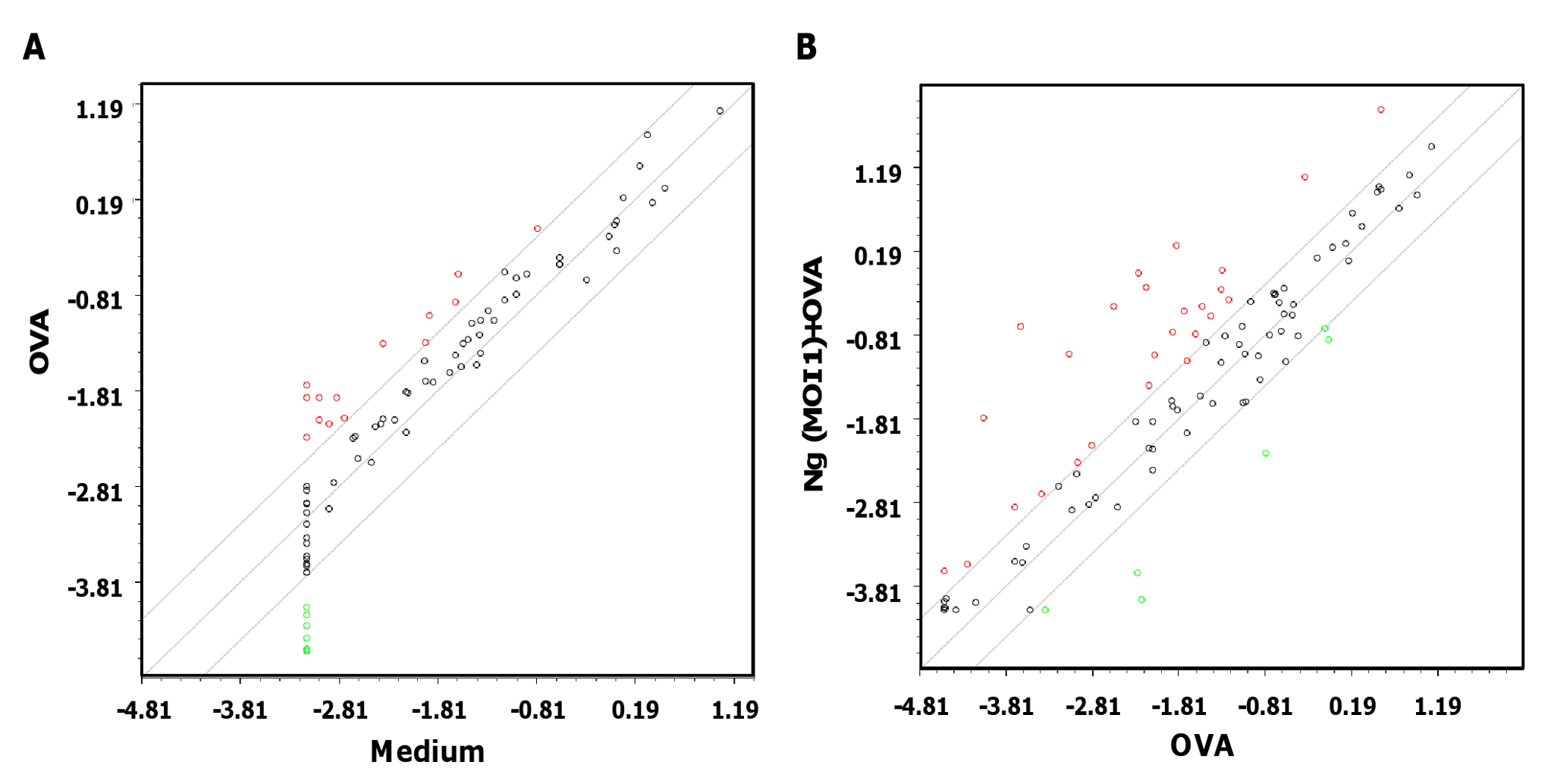

Supplement: Figure S7 — Expression of inflammatory genes was upregulated in BMDCs 24 hours post N. gonorrhoeae exposure. Representative scatter plots of gene expression (qRT-PCR arrays) from A) medium only versus OVA-pulsed BMDCs and B) OVA-pulsed BMDCs versus N. gonorrhoeae (MOI = 1) with OVA, N = 3. (TIFF) [file pone.0041260.s007.tiff]
